# Comparative genomics and transcriptomics analyses provide insights into the high yield and regulatory mechanism of Norvancomycin biosynthesis in *Amycolatopsis orientalis* NCPC 2-48

**DOI:** 10.1186/s12934-021-01521-6

**Published:** 2021-02-02

**Authors:** Xingxing Li, Cong Zhang, Ying Zhao, Xuan Lei, Zhibo Jiang, Xuexia Zhang, Zhihui Zheng, Shuyi Si, Lifei Wang, Bin Hong

**Affiliations:** 1 NHC Key Laboratory of Biotechnology of Antibiotics, Beijing, China; 2 CAMS Key Laboratory of Synthetic Biology for Drug Innovation, Institute of Medicinal Biotechnology, Chinese Academy of Medical Sciences & Peking Union Medical College, No. 1 Tiantan Xili, Beijing, 100050 China; 3 New Drug Research and Development Co. Ltd., North China Pharmaceutical Group, Shijiazhuang, 050015 Hebei China

**Keywords:** Norvancomycin, *Amycolatopsis orientalis*, Comparative transcriptomics, Biosynthetic gene cluster, Transcriptional regulation

## Abstract

**Background:**

Norvancomycin has been widely used in clinic to treat against MRSA (Methicillin-resistant *Staphylococcus aureus*) and MRSE (Methicillin-resistant *Staphylococcus epidermidis*) infections in China. *Amycolatopsis orientalis* NCPC 2-48, a high yield strain derived from *A. orientalis* CPCC 200066, has been applied in industrial large-scale production of norvancomycin by North China Pharmaceutical Group. However, the potential high-yield and regulatory mechanism involved in norvancomycin biosynthetic pathway has not yet been addressed.

**Results:**

Here we sequenced and compared the genomes and transcriptomes of *A. orientalis* CPCC 200066 and NCPC 2-48. These two genomes are extremely similar with an identity of more than 99.9%, and no duplication and structural variation was found in the norvancomycin biosynthetic gene cluster. Comparative transcriptomic analysis indicated that biosynthetic genes of norvancomycin, as well as some primary metabolite pathways for the biosynthetic precursors of norvancomycin were generally upregulated. *AoStrR1* and *AoLuxR1*, two cluster-situated regulatory genes in norvancomycin cluster, were 23.3-fold and 5.8-fold upregulated in the high yield strain at 48 h, respectively. Over-expression of *AoStrR1* and *AoLuxR1* in CPCC 200066 resulted in an increase of norvancomycin production, indicating their positive roles in norvancomycin biosynthesis. Furthermore, AoStrR1 can regulate the production of norvancomycin by directly interacting with at least 8 promoters of norvancomycin biosynthetic genes or operons.

**Conclusion:**

Our results suggested that the high yield of NCPC 2-48 can be ascribed to increased expression level of norvancomycin biosynthetic genes in its cluster as well as the genes responsible for the supply of its precursors. The norvancomycin biosynthetic genes are presumably regulated by AoStrR1 and AoLuxR1, of them AoStrR1 is possibly the ultimate pathway-specific regulator for the norvancomycin production. These results are helpful for further clarification of the holistic and pathway-specific regulatory mechanism of norvancomycin biosynthesis in the industrial production strain.

## Background

**Fig. 1:**
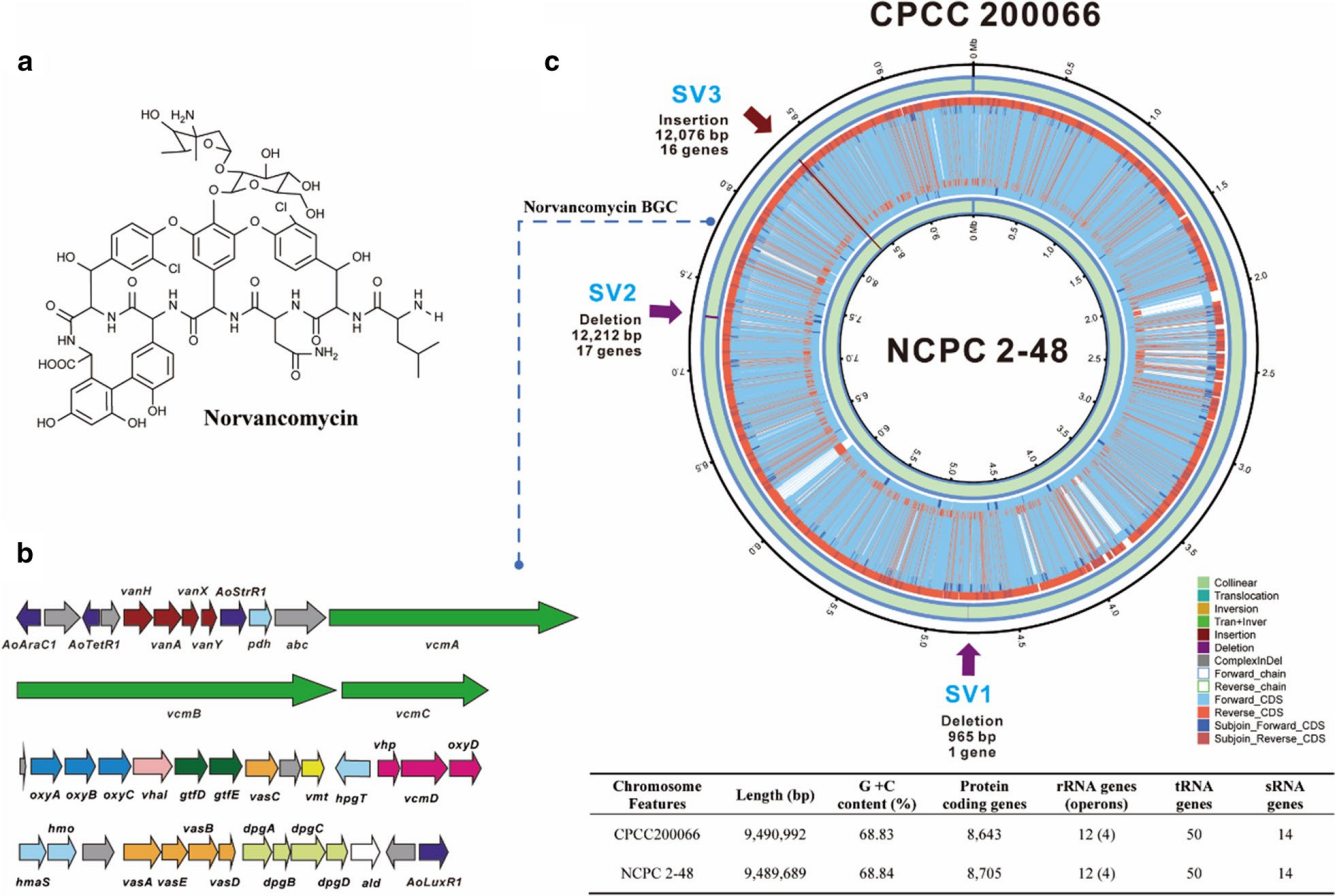
Norvancomycin’s structure, biosynthetic gene cluster and genomic features of its producing strains. **a** Chemical structure of norvancomycin. **b** Organization of the *nvcm* BGC. **c** Comparison of genomic sequences of *A. orientalis* CPCC 200066 and NCPC 2-48. The inner cycle and outer cycle represent the genome of NCPC 2-48 and CPCC 200066, respectively. Delete region (SV1 and SV2) are indicated by purple arrows. Insertion region (SV3) is indicated by brown arrows

Glycopeptide antibiotics, exhibiting outstanding activity against Gram-positive pathogens, are a class of widely known natural compounds produced by Actinomycetes with typical representatives of vancomycin, balhimycin, teicoplanin, A40926 etc. [1]. Some of glycopeptide antibiotics have been approved for clinical use to treat persistent infections by Gram-positive multi-resistant pathogens since vancomycin was first approved in 1958 [2]. Norvancomycin is an important glycopeptide antibiotic of vancomycin group, and has been used as a first-line empiric antibiotic therapy to prevent and treat the intracranial infections of MRSA (Methicillin-resistant *Staphylococcus aureus*) and MRSE (Methicillin-resistant *Staphylococcus epidermidis*) in China since 1969, although its structure was finally determined in the 1980s [3]. Norvancomycin (Fig. 1a) has a closely similar chemical structure to vancomycin and shows a comparable antibacterial spectrum and activity to those of vancomycin. The fermentation potency of norvancomycin in the original strain *Amycolatopsis orientalis* CPCC 200066 was about 200 μg/ml (unpublished data). After series of physical and chemical mutagenesis, a high yield norvancomycin strain (*A. orientalis* NCPC 2-48) was obtained from *A. orientalis* CPCC 200066 by North China Pharmaceutical Group [4]. Using the patented fermentation medium, the fermentation potency of high-yield strain could be increased up to more than 6000 μg/ml. Although *A. orientalis* NCPC 2–48 has been successfully applied to industrial large-scale production, the potential high-yield mechanism and its biosynthetic regulatory mechanism remain obscure.

The biosynthetic gene clusters (BGCs) for some known glycopeptide antibiotics were reported, including vancomycin (*vcm*) [5], balhimycin (*bal*) [6], A40926 (*dbv*) [7], teicoplanin (*tcp*) [8, 9], pekiskomycin (*pek*) [10] and kistamicin (*kis*) [11] etc. In our previous studies, we reported the genome sequence of *A. orientalis* CPCC 200066 and localized the biosynthetic gene cluster of norvancomycin (*nvcm*) [12] which displays a high resemblance with reported gene clusters of *vcm* in *A. orientalis* sub sp. *orientalis* KCTC 9412^ T^ [5]. More than forty genes were identified in the *nvcm* cluster (Fig. 1b), including four resistance proteins, three large NRPSs, several post-assembly tailoring enzymes, and a series of biosynthetic proteins for the supply of non-proteinaceous amino-acid precursors. Combining bioinformatics and biochemical studies, most biosynthetic steps of glycopeptide antibiotics are gradually deciphered [13, 14]. Since chemical structure of norvancomycin is closely similar to vancomycin (Fig. 1a), with only an *N*-methyl group absence at the N-terminus of the polypeptide, we speculate that the biosynthesis of norvancomycin also includes three steps by analogy with vancomycin [15, 16].

The regulatory mechanisms of glycopeptide antibiotics biosynthetic clusters are still not completely characterized. The VanR-VanS pair, a two-component regulatory system which controls the transcription of glycopeptide antibiotics resistance genes [17], have been found in the A40926 cluster (Dbv6 and Dbv22) [7], balhimycin cluster (VnlRS) [18] and teicoplanin cluster (Tei2 and Tei3, also named as Tcp7 and Tcp6) [19]. Nevertheless, the vancomycin and norvancomycin cluster lack VanRS although components of self-resistance system, *e.g.*, *vanHAXY*, are present in the *vcm* and *nvcm* cluster. StrR-family regulators are most commonly present in glycopeptide antibiotics biosynthetic clusters as specific pathway regulators. Bbr in balhimycin cluster binds to five promoter regions within *bal* cluster in vitro, and these genes are responsible for dehydrovancosamine biosynthesis (*dvaA*), oxidative cross-link (*oxyA*), transportation (*tba*), sodium-proton antiporter (*orf7*) and self-regulation (*bbr*) [20]. Likewise, Dbv4 and Tcp28 (also named as Tei15*) were proved to be positive regulators in the A40926 cluster and teicoplanin cluster, respectively [21–23]. Moreover, the proposed palindromic sequence (GTCCAaN_17_TtGGAC) for Bbr-homologous regulator-binding site is also conserved within *bal*, *dbv* and *tcp* BGCs [20, 21, 23]. In addition, LuxR-family regulators have been characterized in A40926 and teicoplanin cluster. Dbv3 positively regulates production of A40926 and hierarchically control the transcription of *dbv4* (StrR-family) [22]. However, unlike StrR-family regulator, the direct target of LuxR-family regulator in the glycopeptide antibiotic clusters remain to be elucidated, since there is no direct binding evidence currently. The bioinformatic analysis indicates that there are four putative regulators (LuxR-family, StrR-family, TetR-family and AraC-family) present in or nearby the *vcm* or *nvcm* cluster. The regulatory mechanism of gene expression for the vancomycin or norvancomycin BGCs has not been reported yet.

The purpose of this study is to uncover the high-yield and biosynthetic regulatory mechanism of industrial strain NCPC 2-48 through exploring the genomic and transcriptomic features compared with the original strain CPCC 200066. Here, we present comprehensive comparative genomics and transcriptomics analyses of these two strains and show that the whole biosynthetic gene cluster of norvancomycin, as well as some primary metabolite pathways for the amino acid and other precursors of norvancomycin were comprehensively upregulated. The cluster situated regulators, AoStrR1 and AoLuxR1, play a positive role in norvancomycin production, and almost all of the norvancomycin biosynthetic genes are directly controlled by AoStrR1. These results have provided insights into the mechanism of high yield production in the industrial strain, as well as the role of cluster-specific regulators in the norvancomycin biosynthesis.

## Results and discussion

### Comparative genomics analysis of norvancomycin producing strains

The complete genome of industrial producing strain *A. orientalis* NCPC 2-48 and that of original strain *A. orientalis* CPCC 200066 [12] are both circular chromosomes of 9.5 Mb with a G+C content of 68.84% (Fig. 1c). The genome analysis of industrial strain showed that it contained 8,705 genes, and the total length of genes was 8,579,274 bp, which makes up to 90.41% of genome. There are 404 tandem repeat sequence (35,253 bp), which makes up to 0.3715% of genome, 325 minisatellite DNAs, 11 microsatellite DNAs, 50 tRNAs, and 12 rRNAs.

We compared the genomes of NCPC 2-48 to CPCC 200066 and found that two genomic sequences are extremely similar with a high identity of 99.97%. There was no large fragment duplication or deletion in the entire genome of NCPC 2-48 compared with CPCC 200066. Moreover, the internal structure of the chromosome and gene order were largely conserved without rearrangements appeared in the genome of NCPC 2-48. Both strains have extremely similar codon usage of 50 tRNA genes. For the norvancomycin biosynthesis, further comparative analysis of secondary metabolism gene clusters showed that no duplication, SVs (structural variations) or InDels (insertions and deletions of small fragments (≤ 50 bp)) were occurred in *nvcm* biosynthetic gene cluster in the industrial strain. It suggested that the high-yield of the industrial strain is not caused by the increased copy number of the *nvcm* biosynthetic gene cluster or gene mutations (SVs or InDels) within the cluster.

The genomic difference between these two strains is mainly accounted for three SVs, including two deletions of 965 bp (SV1) and 12,212 bp (SV2) fragments and one insertion of 12,076 bp (SV3) fragment in strain NCPC 2-48 (Fig. 1c, Additional file 1: Table S3). All these SVs appear at coding DNA sequence (CDS) regions but far away from the *nvcm* biosynthetic gene cluster. There are 34 protein coding genes involved in these fragments of deletion and insertion, including 3 regulators, 1 transporter, 3 transposases, 9 other enzymes and 18 unknown proteins (Additional file 1: Table S3). The coding gene information of the three SVs is shown in Additional file 1: Table S4.

The first structural variation, SV1, occurred in gene B37_4355 (Additional file 1: Table S4). The 965 bp of B37_4355 sequence is deleted in genome of NCPC 2-48. SV2 is a deleted fragment of 12,212 bp in genome of NCPC 2-48, corresponding to the original strain’s chromosome position from gene B37_6566 to B37_6583. There are 17 possible CDSs in this region (Additional file 1: Table S4). The further analysis of the sequence flanking SV2 showed that one *lacI* family transcriptional regulatory gene is located upstream. Usually, *lacI* family regulators control the expression of some key enzymes involved in carbon metabolism, and regulate the transcription of a series of downstream genes including some transcriptional factors [24]. The deletion of genes downstream *lacI* might block the regulation of *lacI* and enhance glucose catabolism of primary metabolism, which is conducive to the precursor sugar synthesis of norvancomycin. SV3 is a 12,076 bp of insertion, which contains 16 possible CDSs (Additional file 1: Table S4). Analysis of function of flanking genes revealed that there are some key enzymes of primary metabolism such as acetyl-CoA dehydrogenase and acetyl-CoA synthetase at downstream of SV3.

In addition to three fragments of deletion and insertion, there are also 216 InDels present in the genome of high-yield strain. The coding regions of some important enzymes related to primary and secondary metabolism together with transcription factors, such as LacI, LysR, TetR, MerR, YebC/PmpR, SARP family proteins and two-component regulators were present in these InDels (Additional file 1: Tables. S5, S6). The InDels in these transcriptional factors may affect the expression of norvancomycin through some unknown regulatory mechanism. However, the function of these transcriptional factors remains unexplored. In these SVs and InDels, there are no functionally known genes directly related to the biosynthetic pathway of norvancomycin and its precursors based on their functional annotation. Thus, the genomic variations of industrial strain would be difficult to give a simple explanation on its high-yield production of norvancomycin. Since some key enzymes of primary metabolism were found in these SVs and InDels, or in flanking segments, we speculated that these genomic mutations may change the metabolic flow by affecting expression of the important enzymes of primary metabolism. Meanwhile, more than ten regulatory genes were detected in SVs and InDels. Along with the loss, insertion or mutation of these regulatory genes, the holistic regulation of primary or secondary metabolism and cell growth may be changed in the high-yield producer strain, which ultimately makes the overall metabolic flow more favorable for the biosynthesis of norvancomycin.

### Transcriptomics profiling at norvancomycin producing strains

**Fig. 2:**
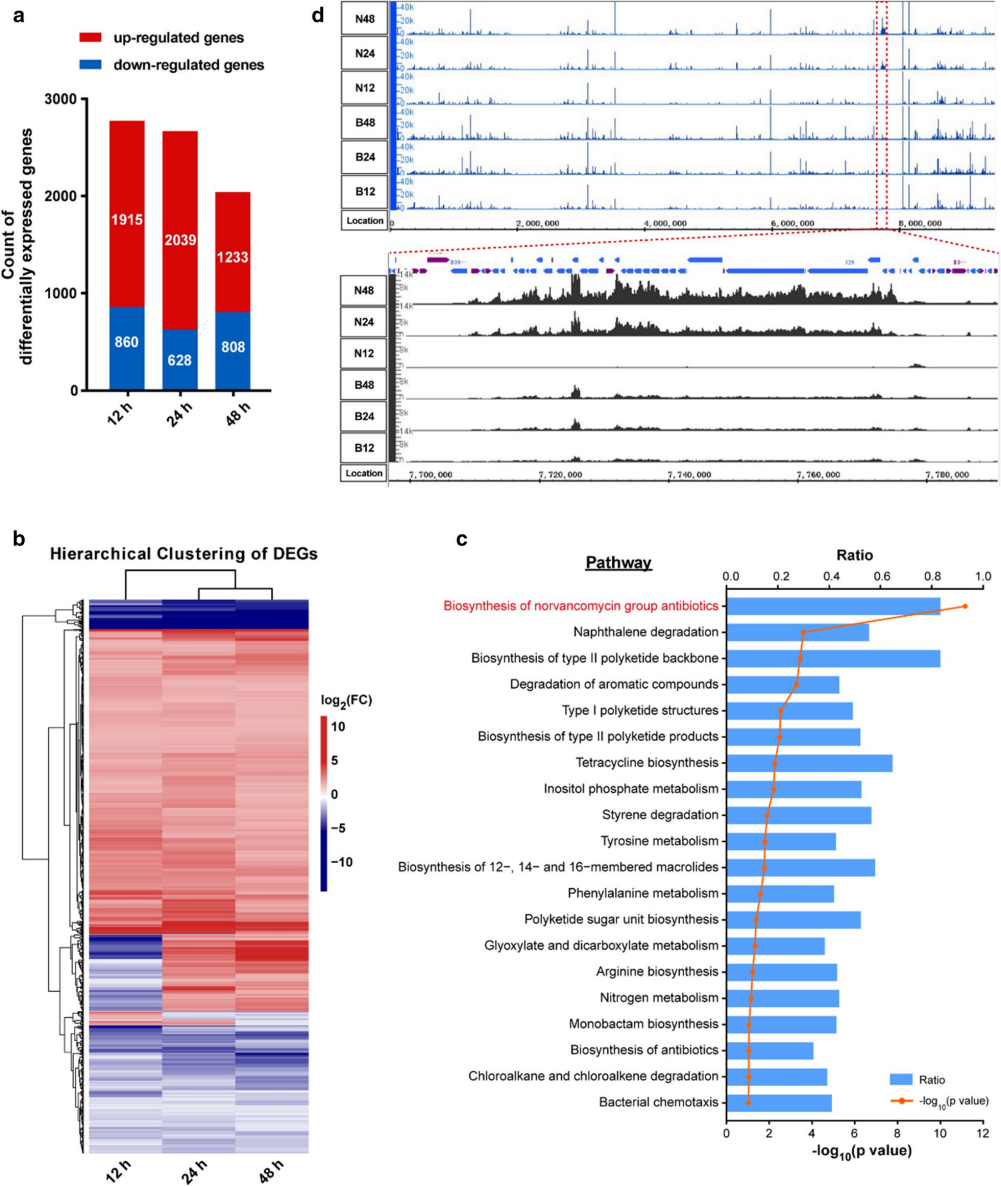
Comparative transcriptomics analysis of *A. orientalis* CPCC 200066 and NCPC 2-48. **a** NCPC 2-48's differentially expressed genes (DEGs) with greater than two-fold change (FC) and FDR ≤ 0.001 in expression compared to the CPCC 200066 at each time points. **b** Hierarchical clustering of DEGs for each time points. Only DEGs that showed in all pairwise were used to build this heatmap. Gradient color barcode at the right top indicates log_2_(fold change) value. Each row represents a gene and each column represents a pairwise. Genes with similar expression value are clustered both at row and column level. **c** Statistics of pathway enrichment of DEGs in 24 h’s pairwise. Ratio is the DEGs numbers annotated in this pathway term to all gene numbers annotated in this pathway term. Greater Ratio means greater intensiveness. Orange line is -log_10_(p-value). We just display the top 20 of enriched pathway terms. **d** The whole genome (top) and *nvcm* cluster (below) expression on transcriptome level for each sample. The samples from the three different time points (12 h, 24 h, 48 h) of the original strain CPCC 200066 were named B12, B24, and B48. The samples of high-yield strains NCPC 2–48 were named N12, N24 and N48

In order to further investigate the mechanism of high yield production of norvancomycin, the transcriptomic analysis of norvancomycin original strain and industrial strain were carried out at three different time points (12 h, 24 h and 48 h). RNA from NCPC 2-48 and CPCC 200066 were extracted and sequenced, and an average of 23,578,714 raw reads were generated. After removing low-quality reads, the average number of remaining clean reads was 23,523,435, and the average comparison rates of clean reads to the reference gene and reference genome were 80.54% and 96.69%, respectively. The statistics on the sequencing data for each sample is shown in Additional file 1: Table S7. Pair-wise differentially expressed gene (DEG) analyses revealed that more than 2000 DEGs, about one-fourth of the total genes in the genome, had significantly lower or higher transcript abundance (fold change (FC) > 2 and false discovery rate (FDR) ≤ 0.001) at each time points (12 h, 24 h and 48 h) in NCPC 2–48 relative to CPCC 200066, as shown in Fig. 2a. The transcriptional levels of some genes within the *nvcm* cluster were verified by RT-qPCR (Additional file 1: Fig. S1). The results of DEGs hierarchical clustering analysis showed that differential gene expression pattern was similar at 24 h and 48 h (Fig. 2b), more than two-thirds of the differential genes were up-regulated in the industrial strain. Interestingly, more genes were downregulated at 12 h compared to other two time points (Fig. 2b), and there are 105 genes downregulated at 12 h but upregulated at 24 h and 48 h time points. KEGG pathway search showed that 35 out of these 105 genes are located in *nvcm* cluster. The biosynthetic pathways of secondary metabolites are usually activated in a growth phase-dependent manner, so that the genes responsible for secondary metabolism coincide with the onset of stationary phase in liquid fermentation in microorganisms. In the case of high-yield strain, transcriptional levels of genes responsible for biosynthesis of norvancomycin are lower in the early stage of growth (12 h), and then upregulated abruptly from 24 to 48 h, showing that the norvancomycin biosynthesis is more strictly controlled during the different growth stages. Due to the similarity of gene expression patterns between 24 h and 48 h, and most of DEGs included at 24 h (2,039 up-regulated and 628 down-regulated), we then analyzed the functional pathway enrichment of DEGs at 24 h based on KEGG database. Enrichment analysis of the functional categories of the transcriptome indicated that a total of 1,764 differential genes were annotated into 150 metabolic pathways, most of them related to the primary metabolism such as nitrogen metabolism and arginine biosynthesis, as well as the biosynthesis of secondary metabolites such as norvancomycin, tetracyclines and other type II polyketides, degradation of naphthalene and aromatic compounds, tyrosine and inositol phosphate metabolism processes. Top 20 of most specific KEGG enrichment results as shown in Fig. 2c.

**Fig. 3:**
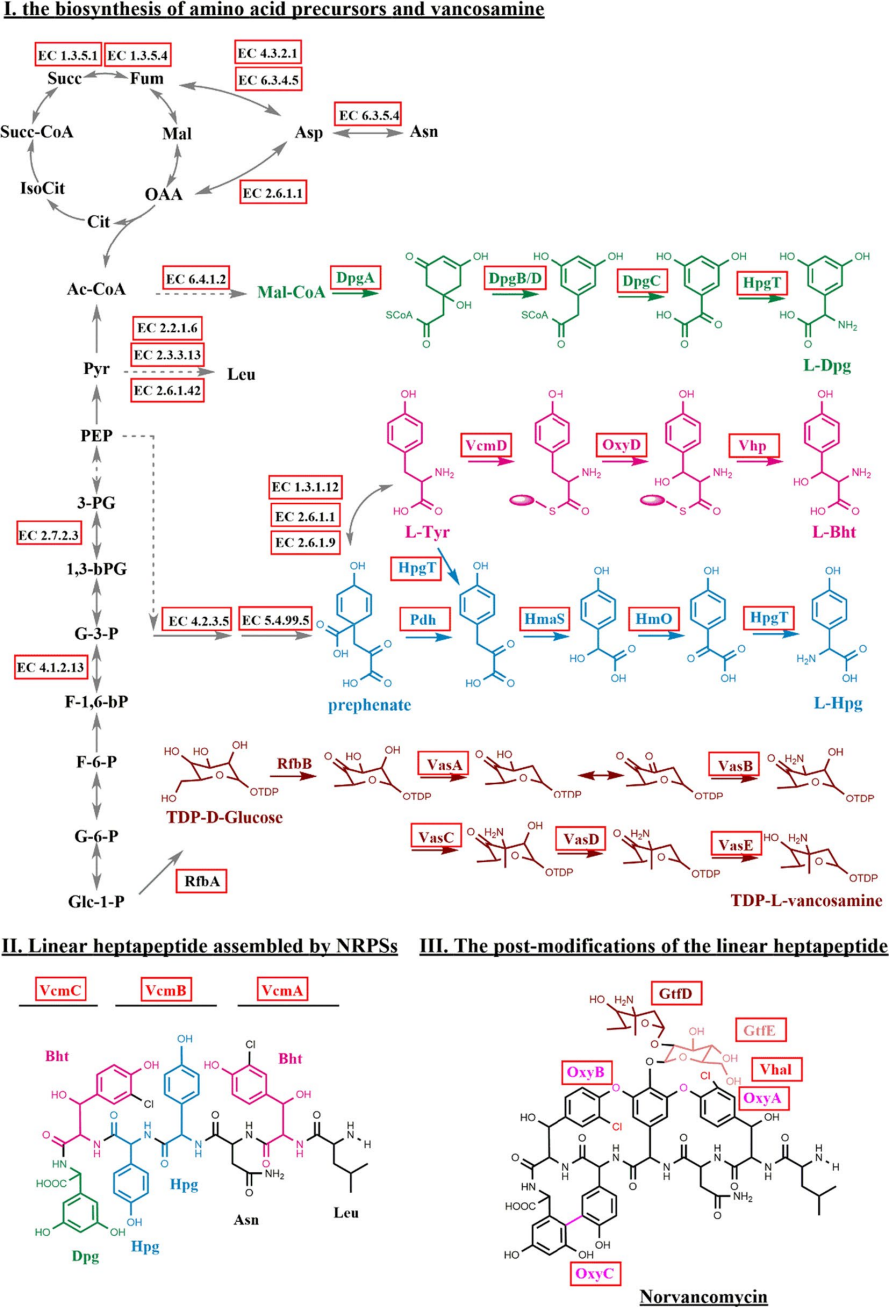
Metabolic pathway of norvancomycin biosynthesis up-regulated in high-yield strain. Three steps are involved in the biosynthesis of norvancomycin: I) the biosynthesis of amino acid precursors, II) The modified amino acids are assembled to form linear heptapeptide by NRPSs, III) The post-modifications of the linear heptapeptide. Red box at each gene indicates that its transcriptional level was up-regulated (> two-fold change and FDR ≤ 0.001) in NCPC 2–48 at 24 h

In particular, the visualization of the transcriptome (Fig. 2d) showed that in the *nvcm* cluster, all key enzymes related to the biosynthesis of respective unit substrates such as Bht (VcmD, OxyD, Vhp), Hpg (Pdh, HmaS, HmO, HpgT), Dpg (DpgA/B/C/D, HpgT) and vancosamine (VasA/B/C/D/E), heptapeptide assemblage (VcmA/B/C) and post-modifications (OxyA/B/C, Vhal, GtfD/E) were transcribed significantly higher in high yield strain than that of original strain at 24 h and 48 h (Fig. 3, Additional file 1: Table S8). It suggested that the increased transcriptional level of norvancomycin's biosynthetic genes (1.9–8.6-fold upregulated at 24 h, and 3.0–18.3-fold upregulated at 48 h) directly promote high yield of norvancomycin in NCPC 2-48. In addition, primary pathways for amino acid (Leu, Asn, Tyr) and glucose production were also upregulated (Fig. 3, Additional file 1: Table S9), including genes B37_4517 and B37_7337 for prephenate (the precursor of Hpg); B37_6997, B37_3479 and B37_6785 for Tyr (the precursor of Bht); B37_7779 for Malonyl-CoA (Dpg's precursor); B37_4701, B37_8154 and B37_2225 for Leu; B37_7110 and B37_3479 for Asn and its precursor Asp synthesis; B37_7117 (RfbA) for TDP-D-Glucose (vancosamine precursor). The results suggested that every step of the whole pathway of the biosynthesis of norvancomycin was significantly upregulated in the industrial production strain, from the abundant supply of amino acid and glucose precursors to the NRPS assembling and the post modification of the glycopeptide antibiotic.

### AoStrR1 and AoLuxR1 positively regulate the biosynthesis of norvancomycin

**Fig. 4:**
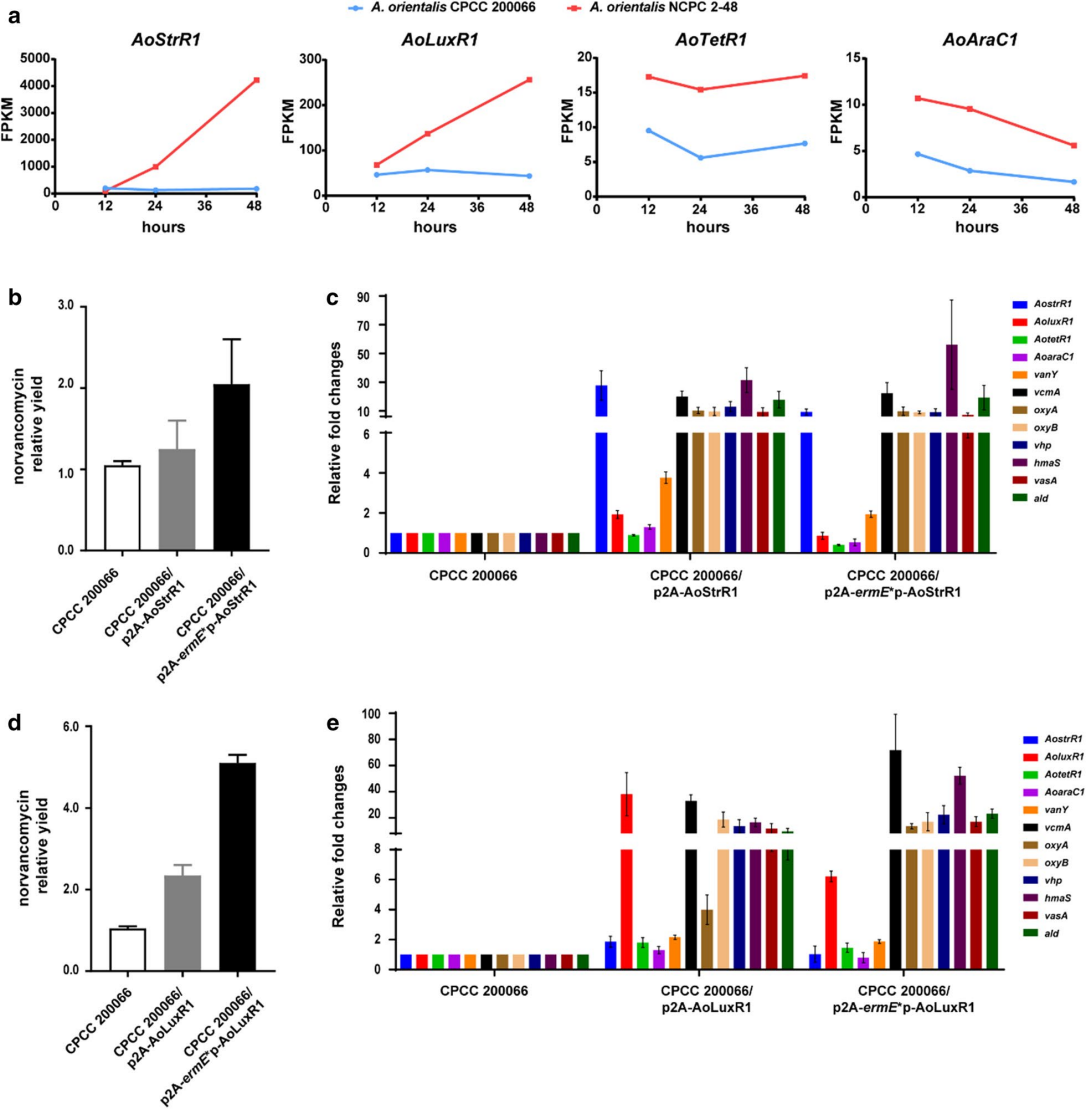
AoStrR1 and AoLuxR1 are cluster-specific positive regulators for norvancomycin biosynthesis. **a** The expression level of four potential regulatory genes at each time points on RNA-seq assay. The gene expression quantification is based on the FPKM (fragments per kilobase of exon per million fragments mapped) value. **b**, **d** Norvancomycin yield in the fermentation product of the original strain CPCC 200066 and AoStrR1/AoLuxR1-overexpressed strains. p2A and p2A-ermE*p represent two overexpressed strains which contain its native promoter or ermE*p strong promoter. Values are presented as means + SEM (two biological repeats for each strain). **c**, **e** Transcriptional analysis of *nvcm* genes in CPCC 200066 and AoStrR1/AoLuxR1-overexpressed strains. The mycelia of each strain were collected at 36 h after fermentation for the extraction of total RNAs, and then these samples were subjected to RT-qPCR analysis. The C_T_ values of the target genes were normalized to the principal sigma factor gene *hrdB*. The relative expression level of each sample was represented as the value related to the sample CPCC 200066. Values are presented as means ± SEM (three biological repeats for each strain)

The expression of antibiotic biosynthetic genes is usually regulated by cluster-specific regulators within the gene cluster. There are four putative regulatory genes (*AoLuxR1*, *AoStrR1*, *AoTetR1*, *AoAraC1*) located within or adjacent to the *nvcm* cluster in the norvancomycin producing strains. The homologues of the four genes also present in a vancomycin producing strain *A. orientalis* KCTC 9412^T^ (Additional file 1: Fig. S2). But in another vancomycin producing strain *A. keratiniphila* HCCB 10007, only homologues of *AoLuxR1, AoStrR1* and *AoTetR1* are present (Additional file 1: Fig. S2). Differential expression analysis revealed that the regulatory genes *AoLuxR1* and *AoStrR1* were significantly upregulated in the high-yield strain*,* with the same trend as the structural genes of *nvcm* clusters (Fig. 4a, Additional file 1: Table S8). The transcriptional levels of these two regulatory genes increased since 24 h and were 23.3-fold and 5.8-fold at 48 h, respectively (Fig. 4a, Additional file 1: Table S8). *AoTetR1* and *AoAraC1* located near the *nvcm* cluster showed 2 ~ 3-fold higher transcription level at 12 h, 24 h and 48 h in the industrial strain than that in the original strain, but in a different trend to the structural genes of *nvcm* clusters, and the transcription level (Fragments Per Kilobase of exon model per Million mapped fragments, FPKM value) were much lower than that of other genes in *nvcm* cluster (Fig. 4a, Additional file 1: Table S8).

The *AoStrR1* gene is located in the *nvcm* cluster (7,787,412–7,788,377 nt), with a total length of 966 bp, encoding the AoStrR1 protein of 321 amino acids. Aligning the amino acid sequences of 22 BGCs of known glycopeptide antibiotics from GenBank (Additional file 1: Table S10) showed that the homologues of AoStrR1 appeared in almost all searched clusters (Additional file 1: Fig. S3A, Table S11), with only exception of corbomycin, the newly discovered compound [25]. These amino acid sequences were used to build phylogenetic tree by MEGA-X program [26] based on Neighbor-Joining method. Compared with the clades of Tcp28 (from teicoplanin BGC), AoStrR1 tended to group with the regulators encoded in BGCs for vancomycin, balhimycin, chloroeremomycin and A40926 (Additional file 1: Fig. S3A). Among them, Bbr and Dbv4, which were confirmed as positive regulators in balhimycin [20] and A40926 [22] cluster respectively, are highly homologous to the AoStrR1 protein with an identity of 84% and 80%, respectively (Additional file 1: Table S11). Thus, we speculated that AoStrR1 might be a pathway-specific regulator of norvancomycin biosynthetic genes, and the increased transcriptional level of *AoStrR1* may be an important factor to trigger production of norvancomycin.

The *AoLuxR1* gene is close to the right border of *nvcm* cluster (7,724,319–7,724,996 nt), with a total length of 678 bp, encoding the AoLuxR1 protein of 225 amino acids. There are 14 possible LuxR-like regulators identified in or adjacent to the 22 glycopeptide BGCs (Additional file 1: Fig. S3B, Table S12). The LuxR phylogenetic tree revealed two main clades (Additional file 1: Fig. S3B). AoLuxR1 appeared to be related to the regulators encoded in BGCs for vancomycin, decaplanin, keratinimicin and nogabecin, with an identity of 83% ~ 98% (Additional file 1: Fig. S3B, Table S12). The other clade included Dbv3 (from A40926 BGC) and Tcp29 (from teicoplanin BGC), which have larger size (more than 500 amino acids) and share low consistency with AoLuxR1 (Additional file 1: Fig. S3B, Table S12). These results suggested that the function or regulatory target of AoLuxR1 may be different from well-characterized Dbv3 or Tcp29. Although the AoLuxR1 homologous gene was not found in the well-known balhimycin BGC in *A. balhimycina* DSM 5908 (there is no adjacent ORF sequences available in GenBank), it is highly conserved in the reported two vancomycin producing strains (Additional file 1: Fig. S2, Table S12).

In order to determine the regulatory function of AoStrR1 and AoLuxR1, we constructed *AoStrR1* and *AoLuxR1* over-expression plasmids based on pULVK2A vector [27], under its native promoter or ermE*p, a strong constitutive promoter, respectively, and then conjugated into *A. orientalis* CPCC 200066. Norvancomycin yield in the fermentation broths of the AoStrR1/AoLuxR1 over-expressing strains were detected by HPLC and LC–MS. Fermentation results showed both AoStrR1 and AoLuxR1 genes could increase norvancomycin production, especially under the ermE*p promoter, which led to a 2 ~ 5 times higher norvancomycin yield (Fig. 4b, d). To confirm the role of AoStrR1 and AoLuxR1 in transcriptional regulation of *nvcm* cluster, the gene expression analysis was conducted by RT-qPCR analysis in over-expression strains. As expected, transcripts of the seven biosynthetic enzyme genes for norvancomycin biosynthesis, *vcmA*, *oxyA*, *oxyB*, *vph*, *hmaS*, *vasA* and *ald*, significantly increased in both over-expression strains (Fig. 4c, e). These results indicated that AoStrR1 and AoLuxR1 both acted as activators for norvancomycin biosynthesis.

### AoStrR1 binds to eight promoter regions in the *nvcm* cluster

**Fig. 5:**
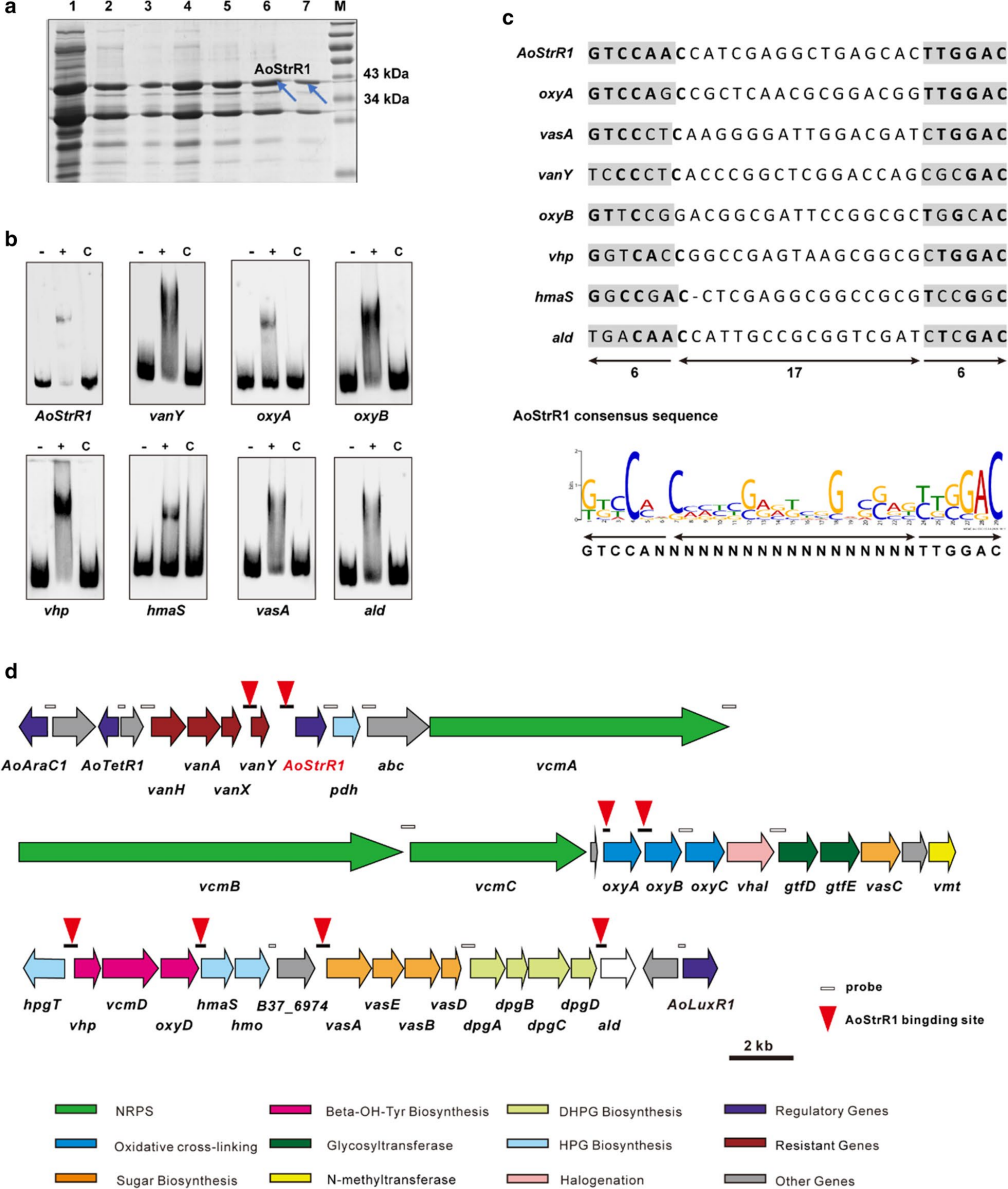
AoStrR1 binds to the promoter regions of the *nvcm* cluster. **a** Overexpression and purification of His_10_-tagged AoStrR1 in *E. coli*. SDS-PAGE showing purification of N-terminal His_10_-tagged AoStrR1 by nickel-affinity chromatography. lanes 1–5, elution with 500 mM imidazole; lanes 6–7, desalted by the PD10 Desalting Columns. M stands for protein size marker. **b** AoStrR1 binds to the 8 promoter regions identified by EMSA. Lane −, probe only; Lane +, 1,000 nM His_10_-AoStrR1 incubated with the probe; Lane C, 1000 nM His_10_-AoStrR1 incubated with 100-fold excess unlabeled specific competitor DNA fragment. **c** Sequence alignment of the putative AoStrR1 binding sites. Consensus sequence was generated using MEME. Arrows indicate inverted repeats and the double-headed arrow denotes the spacer. The most highly conserved nucleotides are bold. **d** The genes present in norvancomycin BGC are indicated by arrows. The short bars are the probes in the *nvcm* cluster. Red triangles are the binding sites of His_10_-AoStrR1

Cluster-specific regulatory proteins generally activate the transcription by binding to the promoter regions of one or more structural genes within a biosynthetic gene cluster, thereby promoting the biosynthesis of secondary metabolites and ultimately increasing their yield. In order to determine the potential target genes of AoStrR1 and AoLuxR1, we tried to express and purify his-tagged AoStrR1 and AoLuxR1 in *E. coli* BL21(DE3), and then perform electrophoretic mobility shift analysis (EMSA). Unfortunately, although different expression conditions were conducted, no soluble His-tagged AoLuxR1 was detected in the supernatant of recombinant *E. coli* BL21(DE3). The soluble AoStrR1 was expressed and purified as a fusion protein with the N-terminal His_10_-tag in *E. coli* BL21(DE3) (Fig. 5a). The expressed protein was checked by western blot with anti-His-tag antibody. As shown in Additional file 1: Fig. S4, a smaller His-tagged protein band was found in addition to the intact His-tagged AoStrR1 and it is likely that a fraction of AoStrR1 is degraded at the C-terminus of the protein. Since the DNA binding domain was predicted present in the C-terminus of AoStrR1, the degraded protein is supposed not be able to bind promoter regions and thus not affect the formation of binding bands between the intact protein and the DNA fragments in subsequent EMSA experiments.

To identify AoStrR1 target genes, 20 intergenic regions in the norvancomycin cluster responsible for the transcription of almost all structural genes and possible regulator genes were amplified and labeled with biotin as probes (200–500 bp) for EMSA experiments. The results showed that the recombinant His_10_-AoStrR1 could form a stable complex with the promoter regions upstream of *vanY*, *AoStrR1*, *oxyA*, *oxyB*, *vhp*, *hmaS*, *vasA* and *ald* (Fig. 5b, d). Addition of 100-fold unlabeled specific competitive DNA attenuated the shift band, indicating that the bindings of His_10_-AoStrR1 to the above probes are specific (Fig. 5b, d). The sequences of above eight AoStrR1 binding sites were input into the GLAM2 [28] and MEME [29] software and the consensus binding sequence of AoStrR1 was identified as GTCCAN_18_TTGGAC containing an incomplete palindromic sequence (Fig. 5c). The AoStrR1 binding motif sequence is highly similar to the reported consensus sequence of its homologues Bbr, Dbv4 and Tcp28 (Tei15*) [20, 21, 23]. This indicated that the StrR-like regulators may have a conservative regulatory mechanism in different glycopeptide BGCs.

Based on RT-qPCR and the EMSA results, AoStrR1 might be the ultimate overall positive regulator responsible for norvancomycin production by binding directly to 8 promoters in *nvcm* cluster, including those of the genes and operons responsible for Bht biosynthesis (Vhp), Hpg biosynthesis (HmaS), vancosamine biosynthesis (VasA), linear heptapeptide cyclization (OxyAB) and self-resistance of norvancomycin (VanY). In addition, AoStrR1 did not bind to the promoters of the other 3 regulators (*AoLuxR1*, *AoTetR1*, *AoAraC1*, Additional file 1: Fig. S5) described here but its own promoter. When comparing the regulatory targets of those reported StrR-like regulators in different glycopeptide BGCs, it is interesting that except for Tcp28 (Tei15*), other three regulators (AoStrR1, Bbr and Dbv4) control one common biosynthetic step, heptapeptide cyclization (*oxyA*), and both AoStrR1 and Bbr could bind to promoter region of genes responsible for specialized amino sugar biosynthesis (*vasA / dvaA*) in their respective gene clusters. Different from Dbv4 and Tcp28 (Tei15*), Bbr and AoStrR1 are able to bind to its own upstream region. It suggested that the positive feedback mechanism of AoStrR1 might be responsible for the significant upregulation of norvancomycin production in the industrial strain. Moreover, among the four StrR-family regulators discussed above, only AoStrR1 could bind to the upstream of *vanY*, part of the putative self-resistance genes. Given that self-resistance regulatory system VanRS is lack in *nvcm* cluster, it is tempting to speculate that AoStrR1 is somehow involved in the regulation of self-resistance.

## Conclusion

In this study, we compared the genomes of original norvancomycin producing strain CPCC 200066 and industrial strain *A. orientalis* NCPC 2–48. Three SVs and some InDels were found in the genome of high-yield strain compared with the original strain, and it is difficult to give a clear clue on its high-yield mechanism of norvancomycin. We further compared the transcriptomes of CPCC 200066 and NCPC 2-48 in three time points to identify differential expression genes involved in the production of norvancomycin. The results showed that the transcriptional upregulation of most *nvcm* biosynthetic genes and genes for precursor supply led to the high yield of norvancomycin in the industrial strain. Furthermore, two positive pathway-specific regulators of norvancomycin production were confirmed by overexpression of AoStrR1 and AoLuxR1. AoStrR1 positively regulates most *nvcm* genes by directly binding to multiple promoter regions in *nvcm* cluster. Thus, this study provides insights into the high yield mechanism and regulatory mechanism of norvancomycin in *A. orientalis* NCPC 2–48, which sets a foundation for future strain improvement.

## Materials and methods

### Strains, plasmids and growth conditions

The norvancomycin-producing strains *A. orientalis* CPCC 200066 and NCPC 2-48 were grown at 28 ℃ on solid Bennet medium (peptone 0.2%, glycerol 1%, beef extract 0.1%, glucose 1%, malt extract 0.3%, yeast extract 0.1%, agar 2%, pH 7.2.) for sporulation and liquid Bennet medium for fermentation. The Mannitol soya flour (MS) medium agar [30] was used for conjugation between *E. coli* and *A. orientalis*. Tryptic soy broth liquid medium [12] was used to grow strains for isolation of genomic DNA. *E. coli* ET12567/pUZ8002 was used for conjugal transfer according to the established protocol [30]. All the strains and plasmids used in this study are listed in Additional file 1: Tables S1 and S2.

### Genomic DNA extraction and sequencing

Genomic DNA were extracted from *A. orientalis* CPCC 200066 and NCPC 2-48 strains using the DNA extraction kit (TANBead, China) according to the manufacturer’s instructions. Genomic DNA library preparation, Illumina sequencing, chromosome assemble and annotation were carried out at Beijing Genomics Institute (Shenzhen, China), and performed as described [12]. The second-generation sequencing platform Illumina Hiseq 2000 and a third-generation sequencing platform Pacbio RSII were used for high-throughput sequencing.

### RNA extraction and sequencing

*RNA extraction.* Strains were collected at 12 h, 24 h and 48 h at the beginning of the fermentation, and total RNA were extracted using the TRIzol reagent (Invitrogen, USA) and chloroform followed by a PureLink™ RNA Mini Kit (Invitrogen, USA) according to the kit’s instructions. The RNA samples from the three different time points of the original strain were named B12, B24, and B48, respectively. The RNA samples of high-yield strains were named N12, N24 and N48, respectively. One biological replicate of each sample was used to perform RNA-seq analysis.

*cDNA library construction and RNA-sequencing.* The above 6 RNA samples were sent to Beijing Genomics Institute (Shenzhen, China) to build cDNA library. The second-generation sequencing platform BGISEQ-500 was used for high-throughput sequencing. After removing low-quality reads, the filtered data was compared with the reference sequence using HISAT [31] and Bowtie2 [32] tools.

*Differentially expressed genes (DEGs).* The RSEM tool [33] was used to quantify gene expression. To eliminate the influence of gene length and sequencing quantity, the results of gene expression quantification were mapped to FPKM (fragments per kilobase of exon per million fragments mapped). We calculated the differential expression of the gene between different samples based on the FPKM value. In our analysis, differentially expressed genes (DEGs) were defined as genes with a fold change more than two times and FDR ≤ 0.001. Hierarchical clustering analysis on DEGs were performed by Cluster [34, 35] and show on javaTreeView [36]. The functional enrichment of DEGs was analyzed by KEGG [37]. The software Integrated Genome Browser [38] was used to create and view the whole genome expression on transcriptome level for each sample.

### Reverse transcription quantitative PCR (RT-qPCR) analysis

Total RNAs were isolated as described above. 2 μg of each of the total RNA was used as a template for reverse transcription (RT), which was performed with the TransScript® One-Step gDNA Removal and cDNA Synthesis SuperMix (Transgen), using random primers following the manufacturer’s instructions. qPCR reaction was detected using CFX96 Touch Real-Time PCR Detection System (Bio-Rad). Each reaction (20 μl) contained 2.5 μl cDNA, 12.5 μl Fast Start Universal SYBR Green Master ROX (Roche) and 0.25 μM of forward and reverse primers (Additional file 1: Table S2). The relative cDNA level of target genes was normalized to the level of *hrdB* according to Pfaffl’s method [39].

### AoStrR1 and AoLuxR1 over-expression in A. orientalis CPCC 200066

The vector pULVK2A [27] and pULVK2A-ermE*p which containing an ermE*p promoter, were used for gene overexpression. The 1310 bp fragment containing the coding region of *AoStrR1* and its native promoter or 1005 bp fragment containing the coding region of *AoLuxR1* and its native promoter were amplified from *A. orientalis* CPCC 200066 genomic DNA by PCR with specific primers (Additional file 1: Table S2), and were cloned into *Hin*dIII-*Bam*HI sites of pULVK2A to obtain the p2A-AoStrR1 and p2A-AoLuxR1. The 981 bp fragment containing coding region of *AoStrR1* or 678 bp fragment containing coding region of *AoLuxR1* were amplified from *A. orientalis* CPCC 200066 genomic DNA by PCR with specific primers (Additional file 1: Table S2) and were cloned into *Nde*I-*Bam*HI sites of pULVK2A-ermE*p to obtain the p2A-ermE*p-AoStrR1 and p2A-ermE*p-AoLuxR1. The constructed vector were introduced into *A. orientalis* CPCC 200066 by intergeneric conjugation from *E. coli* ET12567/pUZ8002 according to the established protocol [40].

### Strain fermentation and norvancomycin yield detection

*Amycolatopsis orientalis* CPCC 200066 and its derivatives were cultured in 100 ml Bennet liquid medium at 28 ℃ for 2 days, and then 10% seed culture was transferred into 100 ml fresh Bennet for continuous fermentation at 28 ℃ for 4 days. Each fermented supernatant was collected by centrifugation at 12,000 rpm for 10 min, and then filtered with microporous membrane of 0.22 μm and analyzed by HPLC–MS (Agilent 1290-Agilent 1956 single quadrupole MS coupled system). The HPLC conditions were as follows: Agilent Eclipase plus C18 column (250 mm × 4.6 mm, 5 μm), mobile phase Solvent A was 100% MeOH, Solvent B was water with 0.1% formic acid. The HPLC program included column elution with a linear gradient of 5 to 30% solvent A over 30 min at 25 ℃. The flow rate was set at 0.8 ml/min. MS spectra data were collected in the positive-ion mode in which a mass range of m/z 150 to 2,000 covered. The NVCM peak was extracted ion chromatogram (EIC) of m/z 717.9 [M + 2H]^2+^.

### AoStrR1 and AoLuxR1 protein expression and purification

The AoStrR1 and AoLuxR1 coding sequence were amplified by PCR from *A. orientalis* CPCC 200066 genomic DNA using specific primers (Additional file 1: Table S2). The amplified DNA fragment were cloned into *Nde*I-*Bam*HI sites of pET-16b (Novagen, Madison, USA) to give pET16b-AoStrR1 or pET16b-AoLuxR1, and then expressed as fusion proteins with the N-terminal His_10_-tag in *E. coli* BL21(DE3). The transformed strains were grown in LB medium until an OD_600_ of 0.8 and induced by adding 0.1 mM IPTG for a further 7 h at 28 ℃. The bacteria were harvested by centrifugation (5000 rpm, 10 min, 4 ℃), and resuspended in 25 ml binding buffer (20 mM NaH_2_PO_4_, 500 mM NaCl, and 20 mM imidazole, pH 7.4), subsequently, lysed by high pressure continuous flow cell cracker (Constant systems, TS 0.75 kW). Cellular debris was removed by centrifugation (12,000 rpm, 20 min, 4 ℃). A HisTrap™ FF crude kit was used to purify recombinant protein, as described by the manufacturer (GE Healthcare). Fractions eluted from the column with 500 mM imidazole were passed through the PD-10 Desalting Columns (GE Healthcare) and eluted with 1 × TGEK buffer (250 mM Tris, 50% Glycerol, 5 mM EDTA, 500 mM KCl, pH 8.0) at 4 ℃, and then stored at − 80 ℃. The concentration of His_10_-tagged AoStrR1 was determined using BCA™ Protein Assay Kit (Pierce Biotechnology, Rockfold, USA) and its purity assessed using SDS-PAGE analysis.

### Electrophoretic mobility shift analysis (EMSA)

DNA fragments containing the promoter regions of the *nvcm* genes were obtained by PCR using primers labeled at their 5′-ends with Biotin (Additional file 1: Table S2) and used as probes in EMSAs. Each 20 μl binding reaction consisted of 2 μl 10× binding buffer (100 mM Tris–HCl, 500 mM KCl, 10 mM DTT, pH 7.5), 2 μg salmon sperm DNA, 20 fmol labeled probe and 1 μM purified His_10_-tagged protein. The specific competitive reactions were carried out by adding 2 pmol competitors (unlabeled probes) apart from ingredients above. Reaction mixtures were incubated at room temperature for 20 min and then analyzed using a native 5% TBE-acrylamide gel and run at 4 °C, 100 V for 80 min. After electrophoretic transfer (15 V for 40 min) to nylon membrane (Hybond-H^+^, GE Amersham) by Trans-BLOT SD Semi-dry transfer cell (Bio-Rad), the probes were visualized by Lightshift Chemiluminescent EMSA Kit (Pierce Biotechnology), according to the manufacturer’s instructions.

### Bioinformatics analyses

Amino acid sequences of proteins homologous to AoStrR1 and AoLuxR1 were retrieved using Blastp tools and were further manually curated to ensure its location within or near the glycopeptide biosynthetic gene cluster. The phylogenetic trees were calculated using the MEGA-X program based on Neighbor-Joining method [26]. The consensus motif of AoStrR1 binding sequence was represented as logos, obtained at the WebLogo website, using GLAM2 [28] and MEME [29] algorithm.

### Data availability

The RNA-sequencing data are publicly available at NCBI’s GenBank’s repository under NCBI BioProject ID: PRJNA624813 (https://www.ncbi.nlm.nih.gov/bioproject/PRJNA624813), BioSample ID: SAMN03273949 & SAMN14585755, SRA ID: SRR11529125 ~ 127 & SRR11529348 ~ 350.

## Supplementary Information

**Additional file 1.** Additional tables and figures.

## Data Availability

All data generated or analyzed during this study are included in this published article and its additional files.
