# Supplementary material for: Comparative genomics and transcriptomics analyses provide insights into the high yield and regulatory mechanism of Norvancomycin biosynthesis in Amycolatopsis orientalis NCPC 2-48
Source: Microb Cell Fact. 2021 Feb 2;20:28. doi: 10.1186/s12934-021-01521-6 (PMC7852140; doi:10.1186/s12934-021-01521-6)
Supplement: Supplementary file 1 — Additional file 1. Additional tables and figures. [file 12934_2021_1521_MOESM1_ESM.docx]

**Supplementary material**

**Comparative genomics and transcriptomics analyses provide insights into the high yield and regulatory mechanism of Norvancomycin biosynthesis in *Amycolatopsis orientalis* NCPC 2-48**

Xingxing Li ^1,2,#^, Cong Zhang ^1,2,#^, Ying Zhao ^3^, Xuan Lei ^1^, Zhibo Jiang ^1^, Xuexia Zhang ^3^, Zhihui Zheng ^3^, Shuyi Si ^1^, Lifei Wang ^1,2 *^, Bin Hong ^1,2 *^

^1^ NHC Key Laboratory of Biotechnology of Antibiotics, Beijing, China

^2^ CAMS Key Laboratory of Synthetic Biology for Drug Innovation, Institute of Medicinal Biotechnology, Chinese Academy of Medical Sciences & Peking Union Medical College, No.1 Tiantan Xili, 100050, Beijing, China.

^3^ New Drug Research and Development Co. Ltd., North China Pharmaceutical Group, 050015, Shijiazhuang, Hebei, China.

^#^ X.L. and C.Z. contributed equally to this work.

^*^ **Correspondence:** Prof. Bin Hong and Prof. Lifei Wang, Institute of Medicinal Biotechnology, Chinese Academy of Medical Sciences & Peking Union Medical College, No.1 Tiantan Xili, 100050, Beijing, China.

**E-mail**: Prof. Bin Hong, binhong69@hotmail.com, hongbin@imb.pumc.edu.cn; Prof. Lifei Wang, lifeiwang2002@hotmail.com, [wanglifei@imb.pumc.edu.cn](mailto:wanglifei@imb.pumc.edu.cn).

**Table of Contents**

[Table S1. Strains and plasmids used in this study. 3](#_Toc60079600)

[Table S2. PCR primers used in this study. 4](#_Toc60079601)

[Table S3 Three SVs in industrial strain *A. orientalis* NCPC 2-48. 6](#_Toc60079602)

[Table S4 The coding gene features in SVs of the original strain *Amycolatopsis orientalis* CPCC 200066 and industrial strain NCPC 2-48. 7](#_Toc60079603)

[Table S5 Summary of InDels in industrial strain *A. orientalis* NCPC 2-48. 9](#_Toc60079604)

[Table S7 Summary of RNA-sequencing data for original strain *A. orientalis* CPCC 200066 and industrial strain NCPC 2-48. 10](#_Toc60079605)

[Table S8 Gene expression in norvancomycin BGC. 11](#_Toc60079606)

[Table S9 Gene expression in primary metabolite pathways for the biosynthetic precursors of norvancomycin. 13](#_Toc60079607)

[Table S10 Characteristics of BGCs for glycopeptide antibiotics. 15](#_Toc60079608)

[Table S11 Mining and comparison of the AoStrR1 homologues in glycopeptide BGCs 16](#_Toc60079609)

[Table S12 Mining and comparison of the AoLuxR1 homologues in glycopeptide BGCs 18](#_Toc60079610)

[Figure S1. Expression levels of five *nvcm* cluster genes detected by RT-qPCR analysis. 19](#_Toc60079611)

[Figure S2. The organization of potential regulators which adjacent to the core biosynthetic genes in four glycopeptide antibiotic clusters. 20](#_Toc60079612)

[Figure S3. Phylogenetic analysis of known and putative StrR-like and LuxR-like transcriptional regulators encoded in glycopeptide BGCs. 21](#_Toc60079613)

[Figure S4. Detection of soluble expression of His_10_-AoStrR1. 22](#_Toc60079614)

[Figure S5. EMSA analysis of the interaction of the promoter regions of the *nvcm* cluster genes with purified His_10_-AoStrR1. 23](#_Toc60079615)

[References 24](#_Toc60079616)

# Table S1. Strains and plasmids used in this study.

| Strains or plasmids | Description | Reference |
| --- | --- | --- |
| **Strains** |  |  |
| *A. orientalis* CPCC 200066 | norvancomycin producing strain (Original strain) | CPCC |
| *A. orientalis* NCPC 2-48 | norvancomycin producing strain (High yield strain) | NCPC |
| *A. orientalis* CPCC 200066/p2A-AoStrR1 | *A. orientalis* CPCC 200066 containing plasmid p2A-AoStrR1，Am^r^ | This work |
| *A. orientalis* CPCC 200066/p2A- *ermE**p-AoStrR1 | *A. orientalis* CPCC 200066 containing plasmid p2A- *ermE**p-AoStrR1，Am^r^ | This work |
| *A. orientalis* CPCC 200066/p2A-AoLuxR1 | *A. orientalis* CPCC 200066 containing plasmid p2A-AoLuxR1，Am^r^ | This work |
| *A. orientalis* CPCC 200066/p2A-*ermE**p-AoLuxR1 | *A. orientalis* CPCC 200066 containing plasmid p2A- *ermE**p-AoLuxR1，Am^r^ | This work |
| *Escherichia coli* DH5α | General cloning host | TransGene |
| *E. coli* ET12567/pUZ8002 | Strain used for *E. coli*/ *A. orientalis* conjugation | [1] |
| *E. coli* BL21(DE3) | Host for the heterologous expression of His_10_-AostrR1 | Novagen |
| **plasmids** |  |  |
| pET-16b | *E. coli* expression vector, Amp^r^ | Novagen |
| pET-16b-AoStrR1 | pET16b derivative plasmid containing 981 bp fragment of *AostrR1* coding region, Amp^r^ | This work |
| pULVK2A | *E. coli*/*A. orientalis* shuttle vector, Am^r^ | [2] |
| p2A-AoStrR1 | pULVK2A derivative plasmid containing 1310 bp fragment of *AoStrR1* coding region under its native promoter, Am^r^ | This work |
| p2A- *ermE**p-AoStrR1 | pULVK2A derivative plasmid containing 981 bp fragment of *AoStrR1* coding region under the *ermE**p strong promoter, Am^r^ | This work |
| p2A-AoLuxR1 | pULVK2A derivative plasmid containing 1005 bp fragment of *AoLuxR1* coding region under its native promoter, Am^r^ | This work |
| p2A- *ermE**p-AoLuxR1 | pULVK2A derivative plasmid containing 678 bp of fragment of *AoLuxR1* coding region under the *ermE**p strong promoter, Am^r^ | This work |

# Table S2. PCR primers used in this study.

| **Name** | **Sequence (5’-3’)^a)^** | **Purpose** |
| --- | --- | --- |
| AoStrR1-F  AoStrR1-R | AAGCTTATTTCCCACGGTGAGACCAC  TATAGATCTTCAGCCGGCGGCCAGATC | Used for amplifying 1,310 bp fragment containing the coding region of AoStrR1 and its native promoter. |
| AoStrR2-F  AoStrR2-R | TATCATATGGATCCGACGAGAGTTGACTATAGATCTTCAGCCGGCGGCCAGATC | Used for amplifying 981 bp coding region of AoStrR1 |
| AoLuxR1-F  AoLuxR1-R | AAGCTTGTGGGGGTGAAATAGAACGA  TATTGATCACTAGACCAGGCCCAGTTC | Used for amplifying 1,005 bp fragment containing the coding region of AoLuxR1 and its native promoter. |
| AoLuxR2-F  AoLuxR2-R | TATCATATGCGGACGAATGACGGCCTC  TATTGATCACTAGACCAGGCCCAGTTC | Used for amplifying 678 bp coding region of AoLuxR1 |
| **RT-qPCR** |  |  |
| HrdB-RTF  HrdB-RTR | TGGTCGAGGTCATCAACAAG  TGGACCTCGATGACCTTCTC | Used for amplifying partial sequence of *hrdB* |
| AoStrR1-RTF  AoStrR1-RTR | CGGTGCACGAGAATAGGC  GAGATCGAGCTGTCCAGGC | Used for amplifying partial sequence of *AostrR1* |
| AoAraC1-RTF  AoAraC1-RTR | CATGACCCTGCCCGAACT  CAGCTCCTTCGCACGATTC | Used for amplifying partial sequence of *AoaraC1* |
| AotetR1-RTF  AotetR1-RTR | GAAACTGCTCGCCGAACTC  TACAGGGAATGGAGCGCC | Used for amplifying partial sequence of *AotetR1* |
| AoluxR1-RTF  AoluxR1-RTR | TACAGCGTCATCACCCAGC  GGCTTCGTCCTTGACCCC | Used for amplifying partial sequence of *AoluxR1* |
| VanY-RTF  VanY-RTR | CTCGATCCGGCTCTCCTC  CTTGGTCTGATACTCCGGTGA | Used for amplifying partial sequence of *vanY* |
| VcmA-RTF  VcmA-RTR | GACGATGAATTCCGCAGCG  CCCGGTAGGTCAGCACCC | Used for amplifying partial sequence of *vcmA* |
| OxyA-RTF  OxyA-RTR | CTGGCGGGTCTCCTCGTAC  GAGCTGCGTGCCCTGATG | Used for amplifying partial sequence of *oxyA* |
| OxyB-RTF  OxyB-RTR | TCACCAGCAGTTCAGCACCC CGGGTCGTAGTCCATCAGGTTG | Used for amplifying partial sequence of *oxyB* |
| Vhp-RTF  Vhp-RTR | CTGATGCAGATCCCATACGG  TCTTACCACGACCAAGGCTC | Used for amplifying partial sequence of *vhp* |
| HmaS-RTF  HmaS-RTR | GACACCGTCGAATCGCCG  AGATCTGCTTGAACCCCAGC | Used for amplifying partial sequence of *hmaS* |
| VasA-RTF  VasA-RTR | TCCTCGGTCCGCATCTGC  TCTTGGCTGGAGCCTCCC | Used for amplifying partial sequence of *vasA* |
| Ald-RTF  Ald-RTR | GAAGGGGCTCATCAACGATC  CCGGAATGGTGGTGTCGA | Used for amplifying partial sequence of *ald* |
| VcmB-RTF  VcmB-RTR | CCAGCAGCGTCTCCCAGG  CGGTTTACGACGGCGAAG | Used for amplifying partial sequence of *vcmB* |
| VcmC-RTF  VcmC-RTR | GCCTGCCACGACCTCTTCA  AAGGGATGCTCTTCCATACCGT | Used for amplifying partial sequence of *vcmC* |
| Vmt-RTF  Vmt-RTR | GCGGCCTCGCCGTAGAAA  TGACGCGTAGCCCGTTGC | Used for amplifying partial sequence of *vmt* |
| **EMSA** |  |  |
| Pdh-1  Pdh-2 | GCCATGCACTCCATCGACGGACAGG  ATCAGCCCGGTCCCGACGACAAGC | Used for amplifying 312 bp promoter region of *pdh* |
| Abc-1  Abc-2 | TGTGGCGGATGATCCTCG  CCGGTCACCTCTCTCGTAAGC | Used for amplifying 378 bp promoter region of *abc* |
| VcmB-1  VcmB-2 | CCTTCGCCGTCGTAAACCG  ACCCGCCAGACCCACTACTCAC | Used for amplifying 289 bp promoter region of *vcmB* |
| VcmC-1  VcmC-2 | GAGCGTTCTAACCCTCATTAGCACT  CAGGACGGTATGGAAGAGCATCCCT | Used for amplifying 296 bp promoter region of *vcmC* |
| OxyA-1  OxyA-2 | AACGGGTGTCCAGCCGCTCAAC  GGTCGAATCGCTCTCTCCGGTG | Used for amplifying 195 bp promoter region of *oxyA* |
| OxyB-1  OxyB-2 | TCACCTGTTCCCTGCTGGCTT  CGGGTCGTCCTCACTCAA | Used for amplifying 399 bp promoter region of *oxyB* |
| OxyC-1  OxyC-2 | CTTCGGGCACGGAGTCCACCATT  TCCGACTGGAAGCTCCTTGGTTGAC | Used for amplifying 310 bp promoter region of *oxyC* |
| GtfD-1  GtfD-2 | CTGAACCCACGGACCCAGGA  CGAACTGCATCTCGACCGTCAT | Used for amplifying 291 bp promoter region of *gtfD* |
| Vhp-1  Vhp-2 | ATCCATGCAGGGTTTCCATGCTCAA  TCCCGTTCTCGGTTGTCATCAGCA | Used for amplifying 436 bp promoter region of *vhp* |
| HmaS-1  HmaS-2 | AGAAGGAACCGGCCTGGTTG  AAAACCCGTACTTGTCCATCCA | Used for amplifying 293 bp promoter region of *hmaS* |
| B37_6974-1  B37_6974-2 | CGGGCTGTGACTCGGTGAGCG  CAAGCCGACCTGGAGCAAGAAGACC | Used for amplifying 167 bp promoter region of *B37_6974* |
| VasA-1  VasA-2 | GGGCAAACTGCTCAGGTCGGACATT  GTACCGGGAGGCTCCAGCCAAGACA | Used for amplifying 350 bp promoter region of *vasA* |
| DpgA-1  DpgA-2 | TACGCCACCAGCCTGGAA  TCTGGTGATCTCGGTCAGTCC | Used for amplifying 231 bp promoter region of *dpgA* |
| Ald-1  Ald-2 | GGGCGTCCGTGCCTTCA  ATCCGGTCGATGCGCTGGTTGT | Used for amplifying 267 bp promoter region of *ald* |
| AoStrR1-1  AoStrR1-2 | GGAGGACAGCCTGGACAGC  CTCAACGTCGAGGACACCC | Used for amplifying 354 bp promoter region of *AostrR1* |
| VanY-1  VanY-2 | GACGAGCCTTACCCGGACACCTACT  GGGCCGTGGTCCGTACTCGTTC | Used for amplifying 404 bp promoter region of *vanY* |
| AoTetR1-1  AoTetR1-2 | ACCGTGCCCCGCAGGGTGACTT  TCCTCCGTGCTCGGGGTGTCGT | Used for amplifying 169 bp promoter region of *AotetR1* |
| AoAraC1-1  AoAraC1-2 | GAACAGGATTCTGATCTTCGGCTTGGC  CCGGTAACCCATCTCCGCATCTTCG | Used for amplifying 257 bp promoter region of *AoaraC1* |
| AoLuxR1-1  AoLuxR1-2 | CGAGTGAGGCCGTCATTCGTCC  CGGTGCGGTGTCGTGAAGGTTC | Used for amplifying 147 bp promoter region of *AoluxR1* |
| VanH-1  VanH-2 | CGGTGCTGGTTCGCTGTAGGTCATG  TGCTGGAGTACGCGGCGTTGTG | Used for amplifying 302 bp promoter region of *vanH* |

^a)^ The restriction sites introduced by these oligonucleotides are underlined.

# Table S3 Three SVs in industrial strain *A. orientalis* NCPC 2-48.

| Features | SV1 | SV2 | SV3 |
| --- | --- | --- | --- |
| Type | Deletion | Deletion | Insertion |
| Length (bp) | 965 | 12212 | 12076 |
| G + C content (%) | 70.98 | 64.25 | 62.01 |
| Total genes | 1 | 17 | 16 |
| Regulators (No.) | 0 | 1 | 2 |
| Transporters (No.) | 0 | 1 | 0 |
| Transposases (No.) | 0 | 2 | 1 |
| Other enzymes (No.) | 1 | 3 | 5 |
| Hypothetical proteins (No.) | 0 | 10 | 8 |

# Table S4 The coding gene features in SVs of the original strain *Amycolatopsis orientalis* CPCC 200066 and industrial strain NCPC 2-48.

| The coding genes in SVs | | |
| --- | --- | --- |
| **SV1** | **Features** | **Type** |
| B37_4355 | α-1,2-mannosidase | Deletion |
| **SV2** | **Features** | **Type** |
| B37_6566 | tyrosine-type recombinase/integrase | Deletion |
| B37_6567 | excisionase | Deletion |
| B37_6568 | replication initiation protein | Deletion |
| B37_6569 | hypothetical protein | Deletion |
| B37_6570 | hypothetical protein | Deletion |
| B37_6571 | hypothetical protein | Deletion |
| B37_6572 | enoyl-CoA hydratase | Deletion |
| B37_6573 | hypothetical protein | Deletion |
| B37_6574 | cell division protein FtsK | Deletion |
| B37_6575 | hypothetical protein | Deletion |
| B37_6576 | hypothetical protein | Deletion |
| B37_6577 | TetR family transcriptional regulator | Deletion |
| B37_6579 | hypothetical protein | Deletion |
| B37_6580 | hypothetical protein | Deletion |
| B37_6581 | ABC transporter | Deletion |
| B37_6582 | hypothetical protein | Deletion |
| B37_6583 | hypothetical protein | Deletion |
| **SV3** | **Features** | **Type** |
| NCPC_7564 | DEAD/DEAH box helicase | Insertion |
| NCPC_7565 | XRE family transcriptional regulator | Insertion |
| NCPC_7566 | hypothetical protein | Insertion |
| NCPC_7567 | XRE family transcriptional regulator | Insertion |
| NCPC_7568 | flavoprotein | Insertion |
| NCPC_7569 | hypothetical protein | Insertion |
| NCPC_7570 | hypothetical protein | Insertion |
| NCPC_7571 | hypothetical protein | Insertion |
| NCPC_7572 | hypothetical protein | Insertion |
| NCPC_7573 | hypothetical protein | Insertion |
| NCPC_7574 | cell division protein FtsK | Insertion |
| NCPC_7575 | cell division protein FtsK | Insertion |
| NCPC_7576 | hypothetical protein | Insertion |
| NCPC_7577 | replication initiator protein | Insertion |
| NCPC_7578 | hypothetical protein | Insertion |
| NCPC_7579 | excisionase | Insertion |

# Table S5 Summary of InDels in industrial strain *A. orientalis* NCPC 2-48.

| Genetic variations | Total number | Gene internal region | | Gene intergenic region | |
| --- | --- | --- | --- | --- | --- |
|  |  | Deletions | Insertions | Deletions | Insertions |
| InDels | 216 | 124 | 27 | 62 | 3 |

# Table S7 Summary of RNA-sequencing data for original strain *A. orientalis* CPCC 200066 and industrial strain NCPC 2-48.

| Sample | Raw reads number | Clean reads number | Clean data rate (%) | reads align to reference genome (%) | reads align to reference gene (%) |
| --- | --- | --- | --- | --- | --- |
| B12 | 23,186,130 | 23,129,836 | 99.75 | 97.02 | 81.89 |
| B24 | 23,175,244 | 23,120,902 | 99.76 | 97.3 | 81.07 |
| B48 | 23,470,380 | 23,388,026 | 99.64 | 97.34 | 77.57 |
| N12 | 23,368,893 | 23,302,663 | 99.71 | 97.59 | 82.23 |
| N24 | 24,135,639 | 24,099,341 | 99.84 | 96.7 | 82.1 |
| N48 | 24,136,002 | 24,099,842 | 99.85 | 94.21 | 78.38 |

# Table S8 Gene expression in norvancomycin BGC.

| gene in CPCC 200066 | gene in NCPC 2-48 | gene name | FPKM | | | | | | Fold Change | | |
| --- | --- | --- | --- | --- | --- | --- | --- | --- | --- | --- | --- |
|  |  |  | B12 | B24 | B48 | N12 | N24 | N48 | N12 vs B12 | N24 vs B24 | N48 vs B48 |
| B37_6962 | NCPC_6996 | AoLuxR1 | 46.53 | 57.02 | 43.98 | 68.35 | 137.10 | 256.39 | 1.47 | 2.40 | 5.83 |
| B37_6963 | NCPC_6997 | AORI_1508 | 25.95 | 29.88 | 43.92 | 9.68 | 162.31 | 372.09 | 0.37 | 5.43 | 8.47 |
| B37_6964 | NCPC_6998 | Ald | 138.04 | 235.39 | 271.13 | 28.84 | 959.42 | 1632.25 | 0.21 | 4.08 | 6.02 |
| B37_6965 | NCPC_6999 | DpgD | 82.38 | 89.61 | 115.55 | 28.79 | 413.14 | 592.73 | 0.35 | 4.61 | 5.13 |
| B37_6966 | NCPC_7000 | DpgC | 150.07 | 110.46 | 158.48 | 17.58 | 608.15 | 911.70 | 0.12 | 5.51 | 5.75 |
| B37_6967 | NCPC_7001 | DpgB | 298.60 | 231.69 | 284.66 | 24.48 | 766.93 | 1270.32 | 0.08 | 3.31 | 4.46 |
| B37_6968 | NCPC_7002 | DpgA | 227.10 | 296.95 | 450.88 | 23.96 | 748.91 | 1668.15 | 0.11 | 2.52 | 3.70 |
| B37_6969 | NCPC_7003 | VasD | 231.42 | 530.78 | 615.60 | 28.93 | 1400.68 | 2495.17 | 0.13 | 2.64 | 4.05 |
| B37_6970 | NCPC_7004 | VasB | 378.72 | 835.16 | 921.65 | 45.69 | 2587.79 | 4311.36 | 0.12 | 3.10 | 4.68 |
| B37_6971 | NCPC_7005 | VasE | 90.75 | 181.82 | 226.94 | 11.14 | 427.17 | 1223.88 | 0.12 | 2.35 | 5.39 |
| B37_6972 | NCPC_7006 | VasA | 148.71 | 357.09 | 351.24 | 19.16 | 967.59 | 2241.82 | 0.13 | 2.71 | 6.38 |
| B37_6973 | NCPC_7007 | - | 41.47 | 38.25 | 64.00 | 8.95 | 326.86 | 676.58 | 0.22 | 8.55 | 10.57 |
| B37_6974 | NCPC_7008 | AORI_1497 | 187.34 | 247.51 | 277.71 | 25.05 | 936.54 | 1793.04 | 0.13 | 3.78 | 6.46 |
| B37_6975 | NCPC_7009 | Hmo | 228.85 | 317.05 | 503.19 | 26.88 | 1097.39 | 2930.12 | 0.12 | 3.46 | 5.82 |
| B37_6976 | NCPC_7010 | HmaS | 1423.50 | 2902.60 | 3762.30 | 102.86 | 5451.92 | 11085.03 | 0.07 | 1.88 | 2.95 |
| B37_6977 | NCPC_7011 | OxyD | 247.96 | 333.86 | 381.96 | 34.38 | 1337.80 | 2521.42 | 0.14 | 4.01 | 6.60 |
| B37_6978 | NCPC_7012 | VcmD | 216.78 | 289.66 | 296.89 | 29.48 | 1205.12 | 2368.91 | 0.14 | 4.16 | 7.98 |
| B37_6979 | NCPC_7013 | Vhp | 229.74 | 297.14 | 346.48 | 31.99 | 1366.50 | 3101.37 | 0.14 | 4.60 | 8.95 |
| B37_6980 | NCPC_7014 | HpgT | 329.26 | 478.15 | 559.00 | 61.13 | 1548.84 | 2839.92 | 0.19 | 3.24 | 5.08 |
| B37_6981 | NCPC_7015 | Vmt | 970.27 | 1478.36 | 1672.20 | 184.34 | 4560.78 | 9074.10 | 0.19 | 3.09 | 5.43 |
| B37_6982 | NCPC_7016 | AORI-1489 | 602.56 | 594.05 | 715.93 | 86.96 | 2955.44 | 5433.52 | 0.14 | 4.98 | 7.59 |
| B37_6983 | NCPC_7017 | VasC | 736.35 | 770.43 | 796.94 | 111.76 | 2937.09 | 4817.59 | 0.15 | 3.81 | 6.05 |
| B37_6984 | NCPC_7018 | GtfE | 407.69 | 550.98 | 536.34 | 67.99 | 1903.33 | 3385.51 | 0.17 | 3.45 | 6.31 |
| B37_6985 | NCPC_7019 | GtfD | 443.94 | 579.48 | 649.51 | 72.18 | 2108.99 | 3784.21 | 0.16 | 3.64 | 5.83 |
| B37_6986 | NCPC_7020 | Vhal | 539.70 | 687.30 | 869.28 | 85.67 | 2429.26 | 4256.76 | 0.16 | 3.53 | 4.90 |
| B37_6987 | NCPC_7021 | OxyC | 184.59 | 205.95 | 279.17 | 33.67 | 929.69 | 2306.06 | 0.18 | 4.51 | 8.26 |
| B37_6988 | NCPC_7022 | OxyB | 148.89 | 134.34 | 156.39 | 38.46 | 577.23 | 1191.14 | 0.26 | 4.30 | 7.62 |
| B37_6989 | NCPC_7023 | OxyA | 249.07 | 268.71 | 351.95 | 60.87 | 1059.00 | 2181.56 | 0.24 | 3.94 | 6.20 |
| B37_6990 | NCPC_7024 | mbtH | 769.99 | 760.44 | 943.28 | 247.27 | 2498.10 | 4537.01 | 0.32 | 3.29 | 4.81 |
| B37_6991 | NCPC_7025 | VcmC | 248.82 | 330.00 | 299.07 | 29.33 | 1095.17 | 2099.29 | 0.12 | 3.32 | 7.02 |
| B37_6992 | NCPC_7026 | - | 236.11 | 279.48 | 249.21 | 24.01 | 917.00 | 1883.76 | 0.10 | 3.28 | 7.56 |
| B37_6993 | NCPC_7027 | VcmB | 265.20 | 311.42 | 325.04 | 44.68 | 1211.60 | 2683.07 | 0.17 | 3.89 | 8.25 |
| B37_6994 | NCPC_7028 | - | 90.13 | 100.61 | 134.53 | 19.34 | 410.79 | 1237.29 | 0.21 | 4.08 | 9.20 |
| B37_6995 | NCPC_7029 | VcmA | 267.88 | 283.41 | 368.15 | 55.30 | 923.71 | 1809.64 | 0.21 | 3.26 | 4.92 |
| B37_6996 | NCPC_7030 | Abc | 686.08 | 679.00 | 857.59 | 108.41 | 2026.06 | 3620.37 | 0.16 | 2.98 | 4.22 |
| B37_6997 | NCPC_7031 | Pdh | 88.92 | 74.77 | 104.52 | 38.34 | 510.34 | 1908.47 | 0.43 | 6.83 | 18.26 |
| B37_6998 | NCPC_7032 | AoStrR1 | 202.16 | 132.97 | 181.22 | 98.31 | 992.60 | 4225.73 | 0.49 | 7.46 | 23.32 |
| B37_6999 | NCPC_7033 | - | 77.36 | 91.85 | 153.61 | 81.48 | 155.88 | 305.20 | 1.05 | 1.70 | 1.99 |
| B37_7000 | NCPC_7034 | VanY | 62.34 | 62.63 | 56.37 | 93.33 | 85.83 | 123.38 | 1.50 | 1.37 | 2.19 |
| B37_7001 | NCPC_7035 | VanX | 294.89 | 201.46 | 329.81 | 520.60 | 232.87 | 255.10 | 1.77 | 1.16 | 0.77 |
| B37_7002 | NCPC_7036 | VanA | 694.86 | 623.15 | 715.50 | 933.76 | 491.97 | 487.57 | 1.34 | 0.79 | 0.68 |
| B37_7003 | NCPC_7037 | VanH | 380.08 | 404.86 | 396.92 | 576.39 | 263.77 | 233.89 | 1.52 | 0.65 | 0.59 |
| B37_7004 | NCPC_7038 | AORI_1470 | 1.30 | 0.29 | 1.80 | 6.56 | 3.16 | 4.05 | 5.05 | 10.90 | 2.25 |
| B37_7005 | NCPC_7039 | AoTetR1 | 9.52 | 5.60 | 7.68 | 17.27 | 15.43 | 17.42 | 1.81 | 2.76 | 2.27 |
| B37_7006 | NCPC_7040 | AORI_1468 | 4.68 | 5.83 | 8.18 | 35.75 | 41.52 | 47.20 | 7.64 | 7.12 | 5.77 |
| B37_7007 | NCPC_7041 | AoAraC1 | 4.67 | 2.87 | 1.66 | 10.69 | 9.54 | 5.60 | 2.29 | 3.32 | 3.37 |

# Table S9 Gene expression in primary metabolite pathways for the biosynthetic precursors of norvancomycin.

| gene in CPCC 200066 | gene in NCPC 2-48 | Features [EC number] | FPKM | | | | | | Fold Change | | |
| --- | --- | --- | --- | --- | --- | --- | --- | --- | --- | --- | --- |
|  |  |  | B12 | B24 | B48 | N12 | N24 | N48 | N12 vs B12 | N24 vs B24 | N48 vs B48 |
| Prephenate biosynthesis pathway | | | | | | | | | | | |
| B37_4517 | NCPC_4561 | chorismate synthase [EC:4.2.3.5] | 10.17 | 5.32 | 11.36 | 2.56 | 20.84 | 23.03 | 0.3 | 3.9 | 2.0 |
| B37_7337 | NCPC_7371 | chorismate mutase [EC:5.4.99.5] | 56.68 | 23.95 | 24.41 | 62.51 | 63.82 | 69.95 | 1.1 | 2.7 | 2.9 |
| Tyrosine biosynthesis pathway | | | | | | | | | | | |
| B37_6997 | NCPC_7031 | prephenate dehydrogenase [EC:1.3.1.12] | 88.92 | 74.77 | 104.52 | 38.34 | 510.34 | 1908.47 | 0.4 | 6.8 | 18.3 |
| B37_3479 | NCPC_3521 | aspartate aminotransferase [EC:2.6.1.1] | 3.81 | 3.03 | 4.03 | 8.56 | 8.34 | 8.28 | 2.2 | 2.8 | 2.1 |
| B37_6785 | NCPC_6817 | histidinol-phosphate aminotransferase [EC:2.6.1.9] | 14.41 | 6.17 | 7.65 | 19.65 | 19.23 | 10.8 | 1.4 | 3.1 | 1.4 |
| Malonyl-CoA biosynthesis pathway | | | | | | | | | | | |
| B37_7779 | NCPC_7832 | acetyl-CoA carboxylase carboxyl transferase subunit alpha [EC:6.4.1.2] | 32.71 | 24.97 | 27.12 | 56.19 | 56.91 | 42.75 | 1.7 | 2.3 | 1.6 |
| Leucine biosynthesis pathway | | | | | | | | | | | |
| B37_8154 | NCPC_8210 | acetolactate synthase I/II/III large subunit [EC:2.2.1.6] | 37.14 | 27.15 | 32.42 | 66.71 | 60.77 | 59.99 | 1.8 | 2.2 | 1.9 |
| B37_2225 | NCPC_2266 | 2-isopropylmalate synthase [EC:2.3.3.13] | 13.15 | 4.4 | 7.1 | 14.65 | 25.91 | 45.78 | 1.1 | 5.9 | 6.4 |
| B37_4701 | NCPC_4745 | branched-chain amino acid aminotransferase [EC:2.6.1.42] | 25.59 | 10.5 | 15.49 | 53.75 | 37.09 | 20.66 | 2.1 | 3.5 | 1.3 |
| Aapartate, Asparagine biosynthesis pathway | | | | | | | | | | | |
| B37_5171 | NCPC_5215 | argininosuccinate synthase [EC:6.3.4.5] | 57.95 | 12.7 | 13.15 | 11.31 | 247.43 | 62.05 | 0.2 | 19.5 | 4.7 |
| B37_5170 | NCPC_5214 | argininosuccinate lyase [EC:4.3.2.1] | 77.05 | 24.16 | 16.83 | 43.16 | 217.34 | 41.14 | 0.6 | 9.0 | 2.4 |
| B37_7110 | NCPC_7145 | asparagine synthase (glutamine-hydrolysing) [EC:6.3.5.4] | 3.37 | 2.67 | 2.32 | 14.21 | 16.56 | 8.54 | 4.2 | 6.2 | 3.7 |
| B37_3479 | NCPC_3521 | aspartate aminotransferase [EC:2.6.1.1] | 3.81 | 3.03 | 4.03 | 8.56 | 8.34 | 8.28 | 2.2 | 2.8 | 2.1 |
| B37_7662 | NCPC_7714 | fumarate reductase, flavoprotein subunit [EC:1.3.5.1 1.3.5.4] | 492.2 | 364.19 | 323.81 | 685.2 | 573.78 | 269.95 | 1.4 | 1.6 | 0.8 |
| B37_7663 | NCPC_7715 | fumarate reductase, iron-sulfur subunit [EC:1.3.5.1 1.3.5.4] | 298.07 | 188.62 | 198.69 | 485.93 | 377.55 | 181.89 | 1.6 | 2.0 | 0.9 |
| TDP-D-Glucose biosynthesis pathway | | | | | | | | | | | |
| B37_7117 | NCPC_7152 | glucose-1-phosphate thymidylyltransferase [RfbA, EC:2.7.7.24] | 0.94 | 1.42 | 0.78 | 4.36 | 7.24 | 3.96 | 4.6 | 5.1 | 5.1 |
| B37_5304 | NCPC_5349 | fructose-bisphosphate aldolase, class II [EC:4.1.2.13] | 7.96 | 2.12 | 1.34 | 1.58 | 5.56 | 3.63 | 0.2 | 2.6 | 2.7 |
| B37_3103 | NCPC_3149 | phosphoglycerate kinase [EC:2.7.2.3] | 395.95 | 202.75 | 191.84 | 358.42 | 422.73 | 392.65 | 0.9 | 2.1 | 2.0 |

| **No.** | **Compound (Cluster)** | **GenBank accession number** | **Organism** |
| --- | --- | --- | --- |
| 1 | Norvancomycin (*nvcm*) | CP016174.1 | *Amycolatopsis orientalis* CPCC 200066 |
| 2 | Vancomycin (*vcm*) | ASJB00000000.1 | *Amycolatopsis orientalis* KCTC 9412^T^ (ATCC 19795, DSM 40040) |
| 3 | Vancomycin (*vcm*) | CP003410.1 | *Amycolatopsis keratiniphila* HCCB 10007 (CGMCC 6023) |
| 4 | Balhimycin (*bal*) | QHHU00000000.1 | *Amycolatopsis balhimycina* DSM 5908 |
| 5 | A40926 (*dbv*) | AJ561198.1 | *Nonomuraea gerenzanensis* ATCC 39727 |
| 6 | A47934 (*sta*) | U82965.2 | *Streptomyces toyocaensis* NRRL 15009 |
| 7 | Teicoplanin (*tcp*) | AJ605139.1 | *Actinoplanes teichomyceticus* ATCC 31121 (NRRL-B16726) |
| 8 | Pekiskomycin (*pek*) | JX026280.1 | *Streptomyces* sp. WAC1420 |
| 9 | Pekiskomycin (*pek*) | KC688274.1 | *Streptomyces* sp. WAC4229 |
| 10 | UK-68,597 (*auk*) | KF192710.1 | *Actinoplanes* sp. ATCC 53533 |
| 11 | Ristocetin (*ris*) | GCA_000749465.3 | *Amycolatopsis lurida* NRRL 2430 |
| 12 | Ristomycin A | CP008953.1 | *Amycolatopsis japonica* MG417-CF17 |
| 13 | Complestatin (*com*) | AF386507.1 | *Streptomyces lavendulae* |
| 14 | Decaplanin | FOPQ00000000.1 | *Amycolatopsis regifaucium* DSM 45072T |
| 15 | Decaplanin | AOHO00000000.1 | *Amycolatopsis decaplanina* DSM 44594 |
| 16 | Avoparcin | MQUQ00000000.1 | *Amycolatopsis coloradensis* DSM 44225 |
| 17 | Nogabecin | MQUP00000000.1 | *Amycolatopsis keratiniphila* subsp. nogabecina FH 1893 |
| 18 | Keratinimicin | LQMT00000000.2 | *Amycolatopsis keratiniphila* NRRL B-24117 (DSM 44409) |
| 19 | Kistamicin (kis) | CP017717.1 | *Nonomuraea* sp. ATCC 55076 |
| 20 | Feglymycin (feg) | KT809366.1 | *Streptomyes* sp. DSM 11171 |
| 21 | Corbomycin | CP029617.1 | *Streptomyces* sp. WAC01529 |
| 22 | Chloroeremomycin | QHKI00000000.1 | *Kibdelosporangium aridum* A82846 |

# Table S10 Characteristics of BGCs for glycopeptide antibiotics.

# Table S11 Mining and comparison of the AoStrR1 homologues in glycopeptide BGCs

|  |  | **BlastP with AoStrR1** | | | |
| --- | --- | --- | --- | --- | --- |
| **Protein homologues** | **Size (aa)** | **Identity (%)** | **Coverage (%)** | **BLAST bit Score** | **E value** |
| AoStrR1_Norvancomycin  [*Amycolatopsis orientalis* CPCC 200066] | 321 |  |  |  |  |
| WP_081736413.1_Vancomycin  [*Amycolatopsis orientalis* KCTC 9412T] | 321 | 99.38 | 100 | 622 | 0 |
| AGM04063.1_Vancomycin  [*Amycolatopsis keratiniphila* HCCB 10007] | 286 | 92.20 | 87 | 518 | 0 |
| WP_007034155.1_Decaplanin  [*Amycolatopsis decaplanina* DSM 44594] | 324 | 93.15 | 100 | 594 | 0 |
| AIG79246.1_Ristomycin A  [*Amycolatopsis japonica* MG417-CF17] | 265 | 92.80 | 82 | 484 | 2.00E-175 |
| KFU79350.1_Ristocetin  [*Amycolatopsis lurida* NRRL 2430] | 321 | 91.56 | 99 | 579 | 0.00E+00 |
| SFH60749.1_Decaplanin  [*Amycolatopsis regifaucium* DSM 45072T] | 321 | 90.03 | 100 | 577 | 0 |
| WP_076161077.1_Avoparcin  [*Amycolatopsis coloradensis* DSM 44225] | 318 | 88.33 | 98 | 546 | 0 |
| OLZ50883.1_Nogabecin  [*Amycolatopsis keratiniphila* subsp. nogabecina FH 1893] | 321 | 85.94 | 99 | 549 | 0 |
| ONF65144.1_Keratinimicin  [*Amycolatopsis keratiniphila* NRRL B-24117] | 321 | 85.94 | 99 | 549 | 0 |
| Bbr_Balhimycin  [*Amycolatopsis balhimycina* DSM 5908] | 321 | 84.11 | 100 | 533 | 0 |
| RSM88020.1_Chloroeremomycin  [*Kibdelosporangium aridum* A82846] | 321 | 82.87 | 100 | 523 | 0 |
| Dbv4_A40926  [*Nonomuraea gerenzanensis* ATCC 39727] | 321 | 80.06 | 100 | 497 | 0 |
| Pek7_Pekiskomycin  [*Streptomyces* sp. WAC1420] | 321 | 80.00 | 99 | 501 | 0 |
| AGO98976.1_Pekiskomycin  [*Streptomyces* sp. WAC4229] | 321 | 78.75 | 99 | 498 | 0 |
| AQZ71349.1_Kistamicin  [*Nonomuraea* sp. ATCC 55076] | 273 | 60.22 | 82 | 281 | 6.00E-95 |
| StaQ_A47934  [*Streptomyces toyocaensis* NRRL 15009] | 333 | 55.66 | 95 | 322 | 1.00E-112 |
| AAK81822.1_Complestatin  [*Streptomyces lavendulae*] | 348 | 51.10 | 93 | 291 | 3.00E-100 |
| AGS77330.1_UK-68,597  [*Actinoplanes* sp. ATCC 53533] | 329 | 49.22 | 95 | 286 | 2.00E-98 |
| Tcp28(Tei15*)_Teicoplanin  [*Actinoplanes teichomyceticus* ATCC 31121] | 329 | 49.06 | 95 | 279 | 9.00E-96 |
| FegB_Feglymycin  [*Streptomyes* sp. DSM 11171] | 335 | 46.91 | 92 | 251 | 1.00E-84 |

# Table S12 Mining and comparison of the AoLuxR1 homologues in glycopeptide BGCs

|  |  | **BlastP with AoLuxR1** | | | |
| --- | --- | --- | --- | --- | --- |
| **Protein homologues** | **Size (aa)** | **Identity (%)** | **Coverage (%)** | **BLAST bit Score** | **E value** |
| AoLuxR1_Norvancomycin  [*Amycolatopsis orientalis* CPCC 200066] | 225 |  |  |  |  |
| WP_051174007.1_Vancomycin  [*Amycolatopsis orientalis* KCTC 9412T] | 225 | 98.22 | 100 | 431 | 9.00E-157 |
| AGM04097.1_Vancomycin  [*Amycolatopsis keratiniphila* HCCB 10007] | 209 | 84.43 | 94 | 350 | 6.00E-125 |
| KFU79315.1_Ristocetin  [*Amycolatopsis lurida* NRRL 2430] | 215 | 82.95 | 96 | 350 | 8.00E-125 |
| AIG79207.1_Ristomycin A  [*Amycolatopsis japonica* MG417-CF17] | 215 | 84.33 | 96 | 354 | 2.00E-126 |
| SFH61781.1_Decaplanin  [*Amycolatopsis regifaucium* DSM 45072T] | 225 | 90.22 | 100 | 386 | 4.00E-139 |
| WP_039923829.1_Decaplanin  [*Amycolatopsis decaplanina* DSM 44594] | 218 | 87.10 | 96 | 375 | 1.00E-134 |
| OLZ50912.1_Nogabecin  [*Amycolatopsis keratiniphila* subsp. nogabecina FH 1893] | 215 | 84.79 | 96 | 357 | 1.00E-127 |
| ONF72098.1_Keratinimicin  [*Amycolatopsis keratiniphila* NRRL B-24117] | 215 | 84.79 | 96 | 357 | 1.00E-127 |
| Dbv3_A40926  [*Nonomuraea gerenzanensis* ATCC 39727] | 867 | 40.00 | 28 | 21.6 | 1.60E+00 |
| Tcp29(Tei16*)_Teicoplanin  [*Actinoplanes teichomyceticus* ATCC 31121] | 792 | 38.89 | 15 | 18.5 | 4.30E-01 |
| AGS77334.1_UK-68,597  [*Actinoplanes* sp. ATCC 53533] | 807 | 29.17 | 24 | 15.8 | 2.60E+00 |
| AQZ69241.1_Kistamicin  [*Nonomuraea* sp. ATCC 55076] | 556 | 61.54 | 15 | 17.7 | 4.40E-01 |
| FegF_Feglymycin  [*Streptomyes* sp. DSM 11171] | 969 | 75 | 3 | 14.2 | 9.90E+00 |


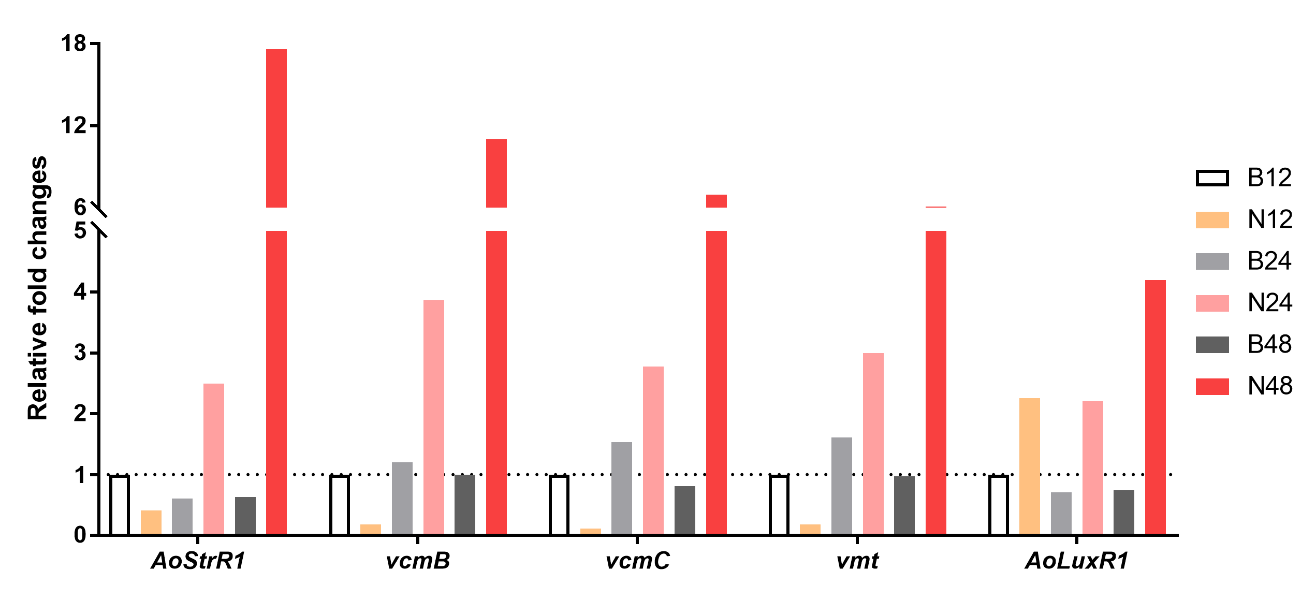


# Figure S1. Expression levels of five *nvcm* cluster genes detected by RT-qPCR analysis.

The mycelia of *A. orientalis* CPCC 200066 and NCPC 2-48 at the early stage of fermentation (12 h, 24 h and 48 h, respectively) were collected for the extraction of total RNAs, and then these samples were subjected to RT-qPCR analysis. The samples from the three different time points (12 h, 24 h, 48 h) of the original strain CPCC 200066 were named B12, B24, and B48. The samples of high-yield strains NCPC 2-48 were named N12, N24 and N48. The C_T_ values of the target genes were normalized to the principal sigma factor gene *hrdB*. The relative expression level of each sample was represented as the value related to the sample B12.

**
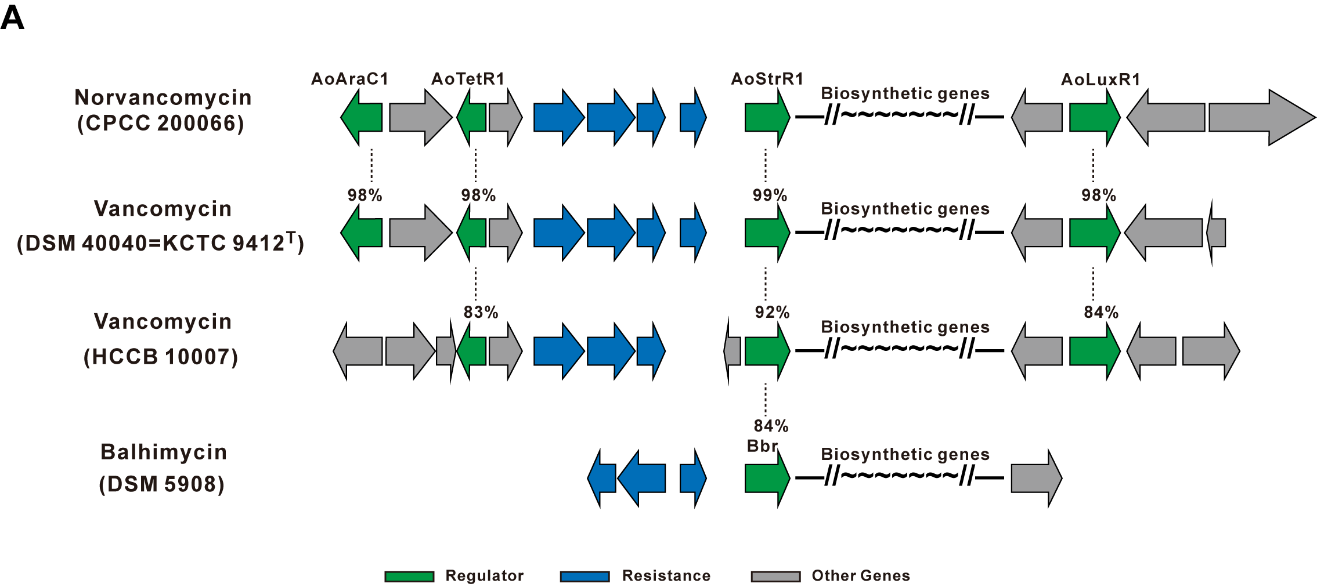
**

# Figure S2. The organization of potential regulators which adjacent to the core biosynthetic genes in four glycopeptide antibiotic clusters.

Norvancomycin producing strain *A. orientalis* CPCC 200066, vancomycin producing strain *A. orientalis* DSM 40040 = KCTC 9412^T^ and *A. keratiniphila* HCCB 10007, balhimycin producing strain *A. balhimycina* DSM 5908. The number above the gene represents the identities of amino acid sequence compared with the homologous protein in CPCC 200066.


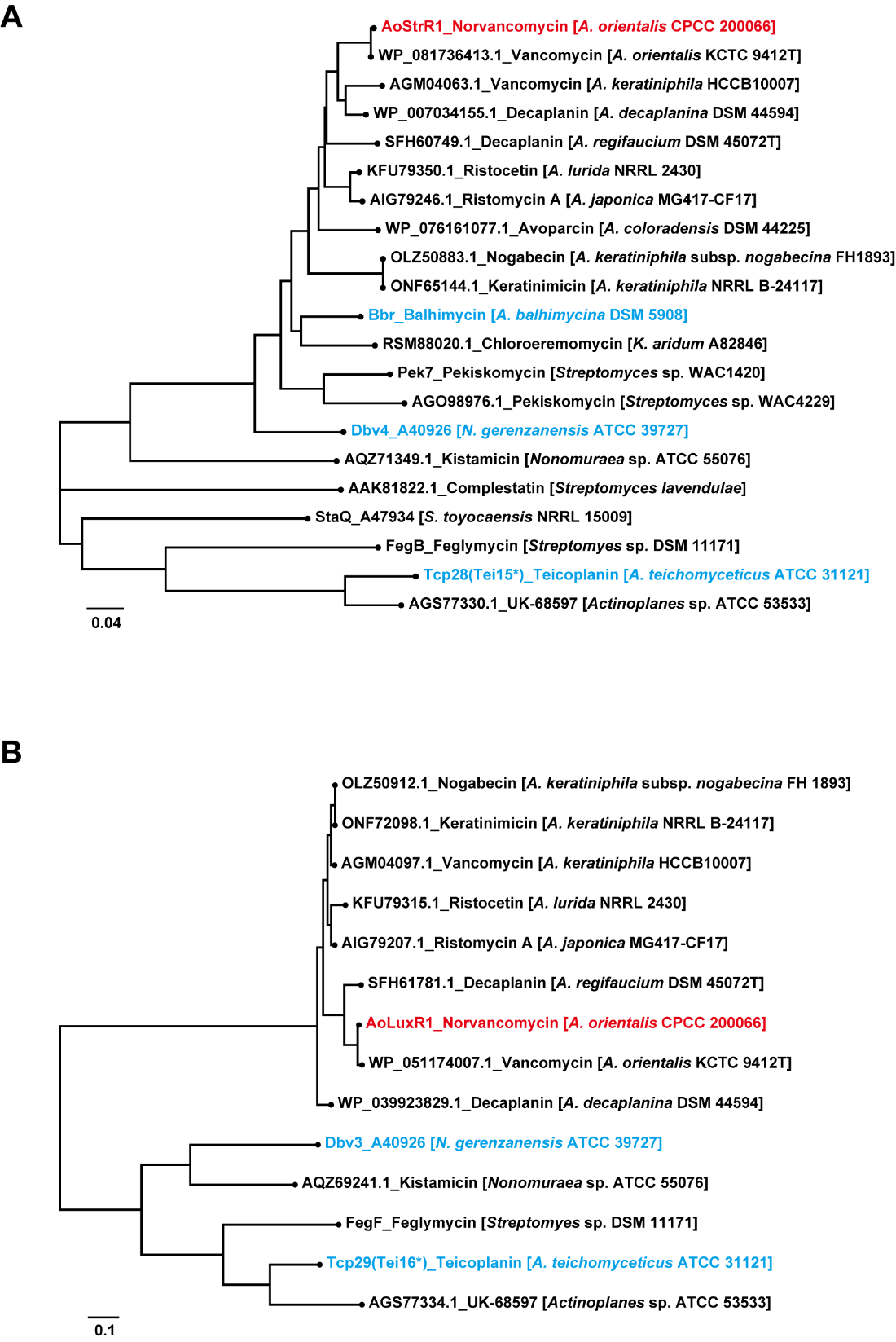


# Figure S3. Phylogenetic analysis of known and putative StrR-like and LuxR-like transcriptional regulators encoded in glycopeptide BGCs.

The AoStrR1 (**A**) and AoLuxR1 (**B**) in norvancomycin BGC are labeled in red. Previous experimentally studied regulators are labeled in blue. MEGA-X program was used to build phylogenetic tree and the final topology was derived from 1,000 bootstraps based on Neighbor-Joining method.


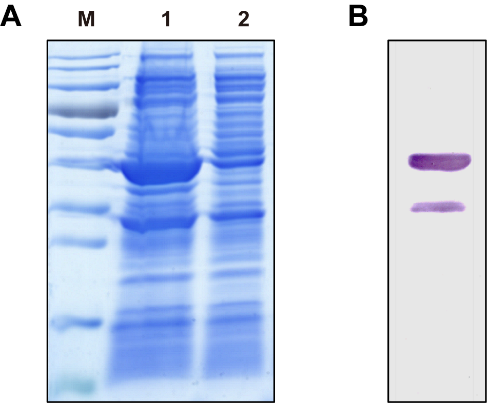


# Figure S4. Detection of soluble expression of His_10_-AoStrR1.

(**A**). SDS-PAGE analysis of the protein expression in recombinant *E. coli* BL21(DE3)/pET16b-AoStrR1 induced by IPTG in LB medium. Lane M, PageRuler Prestained Protein Ladder (170, 130, 100, 70, 55, 40, 35, 25, 15, 10 kDa); Lane 1, the lysate of *E. coli* BL21(DE3)/pET16b-AoStrR1; Lane 2, supernatant of the lysate of *E. coli* BL21(DE3)/pET16b-AoStrR1; (**B).** The supernatant of the lysate of recombinant *E. coli* BL21(DE3)/pET16b-AoStrR1 were analyzed by western-blotting with anti-His-Tag antibody.


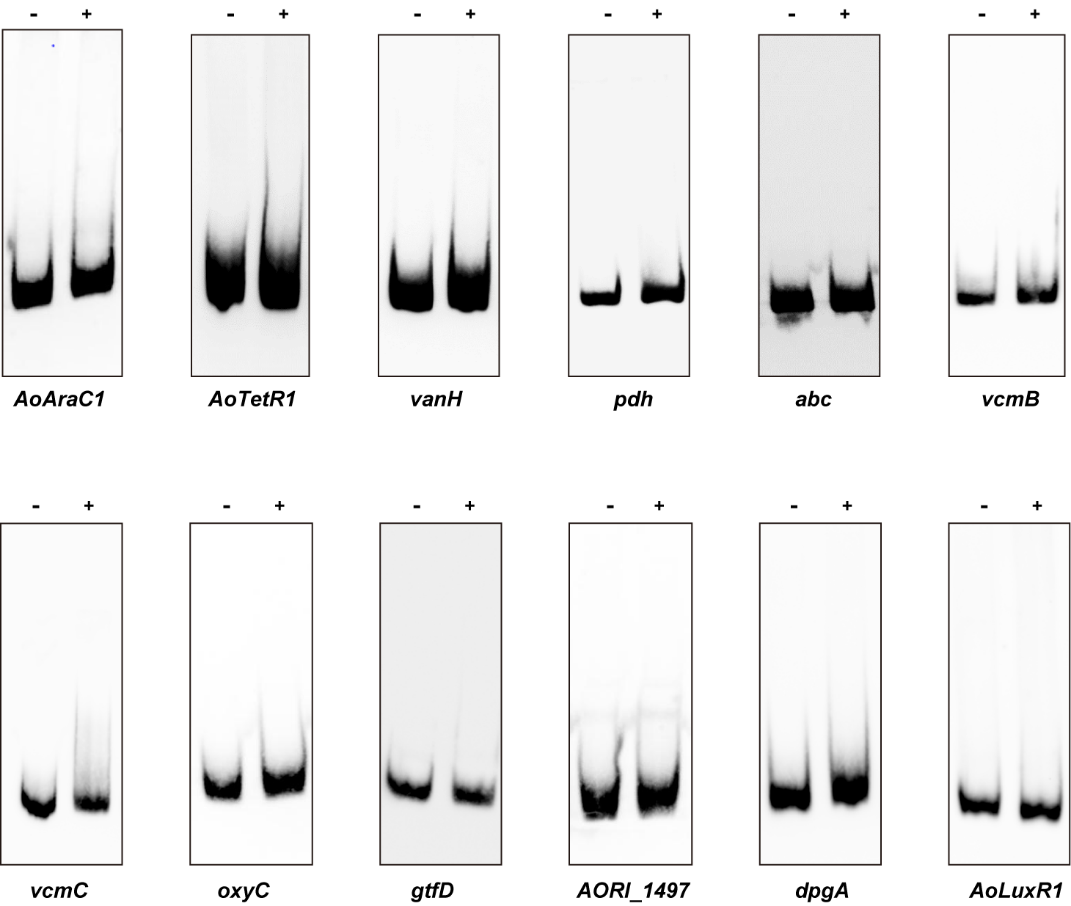


# Figure S5. EMSA analysis of the interaction of the promoter regions of the *nvcm* cluster genes with purified His_10_-AoStrR1.

His_10_-AoStrR1 could not bind to the promoter regions of these 12 genes identified by EMSA. Lane -, probe only; Lane +, 1,000 nM His_10_-AoStrR1 incubated with the probe.

# References

1. Kieser T, Bibb M, Buttner M, Chater K, Hopwood D: Pratical *Streptomyces* Gnetics*.* Norwich: The John Innes Foundation; 2000.

2. Kumar CV, Coque JJ, Martin JF: Efficient Transformation of the Cephamycin C Producer *Nocardia lactamdurans* and Development of Shuttle and Promoter-Probe Cloning Vectors**.** Appl Environ Microbiol. 1994; 60**:**4086-93.
